# Secondary Metabolites from the Marine Sponges of the Genus *Petrosia*: A Literature Review of 43 Years of Research

**DOI:** 10.3390/md19030122

**Published:** 2021-02-25

**Authors:** Yeon-Ju Lee, Yeonwoo Cho, Huynh Nguyen Khanh Tran

**Affiliations:** 1 Marine Natural Products Chemistry Laboratory, Korea Institute of Ocean Science and Technology, 385 Haeyangro, Busan 49111, Korea; yeonwoo@kiost.ac.kr (Y.C.); hnktran@kiost.ac.kr (H.N.K.T.); 2 Department of Applied Ocean Science, University of Science and Technology, Daejeon 34113, Korea

**Keywords:** *Petrosia*, metabolite, geography, polyacetylene, sponge

## Abstract

Sponges are prolific sources of various natural products that have provided the chemical scaffolds for new drugs. The sponges of the genus *Petrosia* inhabit various regions and contain a variety of biologically active natural products such as polyacetylenes, sterols, meroterpenoids, and alkaloids. This review aims to provide a comprehensive summary of the chemical structures and biological activities of *Petrosia* metabolites covering a period of more than four decades (between 1978 and 2020). It is also described in this review that the major groups of metabolites from members of the genus *Petrosia* differed with latitude. The polyacetylenes were identified to be the most predominant metabolites in *Petrosia* sponges in temperate regions, while tropical *Petrosia* species were sources of a greater variety of metabolites, such as meroterpenoids, sterols, polyacetylenes, and alkaloids.

## 1. Introduction

The oceans represent the largest habitat on earth and contain organisms with high biological and chemical diversity. The secondary metabolites of diverse marine organisms allowed these species to develop mechanisms that would enable them to survive in the ocean. Over 30,000 natural products have been isolated from marine organisms [1], and 1490 and 1554 new compounds were discovered recently as 2017 and 2018, respectively [2,3].

Marine invertebrates, such as sponges, cnidarians, bryozoans, echinoderms, and tunicates, exclusively inhabit aquatic environments [4]. They are either sessile or slow moving, have soft bodies, and lack morphological defense structures such as shells or thorns [5]. Therefore, it is not incidental that these organisms evolved chemical defense mechanisms against predation and overgrowth of fouling organisms [6,7]. The abundance and diversity of secondary metabolites synthesized for chemical defense provide an opportunity to examine the chemical entities with potential therapeutic applications.

Marine sponges (*Porifera*) have been the central focus of the research for the discovery of biologically-active secondary metabolites. As sponges are highly effective filter feeders, microorganisms in the surrounding water are actively swirled by the sponge-driven currents. While some of the microorganisms are immediately digested, others are retained within the sponge body. It was reported that associated microorganisms can account for up to 60% of the fresh weight of marine sponges, and are more diverse than can be estimated using current technology [8,9]. It is believed that these sponge-associated microorganisms such as bacteria, fungi, cyanobacteria, and unicellular algae may be involved in the biosynthesis of natural products that are isolated from the sponges, although the relationship between sponge and associated microorganisms are highly complex [10].

Several statistical studies on marine natural products have revealed that sponges are outstanding in number of isolated biologically-active compounds. Hu et al. have analyzed the temporal trend, chemical structure distribution, bioactivity groups, and species distribution of biologically-active compounds from marine organisms discovered during the 28 years from 1985 to 2012 [11]. In this study, the authors found out that approximately 75% of the compounds were isolated from marine invertebrates, and the highest proportion of bioactive compounds had been isolated from sponges. Another report, which covers 9812 marine natural products isolated from invertebrates discovered during 1990–2009, also showed that the highest proportion of metabolites could be traced to sponges (48.8%) [12].

It is generally believed that there is a higher chance of discovering biologically-active sponge metabolites in habitats characterized by intense competition for survival due to their high biological diversity and density, such as coral reefs in tropical regions [13,14]. Following the principle that sponge chemical defense is mainly driven by predation pressure [15], it was hypothesized that chemical diversity is higher in the tropical region than the temperate region. This hypothesis is only partly supported, as it was reported that tropical and temperate benthic systems differ with regard to dominant consumption regimes. While fishes are dominant predators on reefs in tropical regions, temperate systems are characterized by mesopredators such as crustaceans, sea urchins, and gastropods [16,17].

Geographical location is clearly one of the most influential factors related to the variation in sponge metabolites; however, few studies have investigated the geographical variation in sponge metabolites in terms of yield or biological activity. The first study regarding this issue claimed the inverse relationship between latitude and toxicity in sponges [18]. The authors examined the toxicity of 78 sponge species collected in temperate (San Juan Island and Santa Catalina Island) and tropical regions (La Blanquilla reef and Zihuatanejo Bay) on fishes by measuring the time from exposure to death. They found that 9% of the sponge species in the regions of 48 degrees north were toxic and 75% of the sponges studied in the region of 19 degrees north were toxic. A study by Ruzicka et al. also demonstrated that sponge species on the temperate reefs in the South Atlantic Bight are a smaller chemical deterrent to fish predators than their counterparts at lower latitudes in the Florida Keys by measuring the palatability of sponge crude extracts quantitatively and comparing the results with those obtained by Pawlik et al. [14,19].

Results that are contradictory to those above have also been reported. A feeding assay with 17 pairwise comparisons conducted by Burns et al. showed no significant differences in deterrence between Red Sea and Caribbean sponges [20]. The field-based feeding experiments with diverse fish predators conducted by Becerro et al. showed similar results [21]. When 20 sponge species from tropical Guam and temperate Northeast Spanish coasts were compared, no significant difference was observed based on habitat. It is worthwhile to mention, however, that the percentage yield of crude organic extracts tends to be proportional to latitude, although the authors did not provide any comment on this. When a total of 20 sponge species in 10 species pairs were extracted, the tropical sponges were found to provide up to a 3.4 times higher yield than their temperate counterparts in nine pairs.

More recent studies focused on the intra-specific variability in secondary metabolites in terms of concentration. One such study examined *Aplysina aerophoba*, a common Mediterranean sponge known to contain large concentrations of brominated alkaloids [22]. When the concentrations of bromotyrosine alkaloids such as aerophobin-1, aerophobin-2, aplysinamisin-1, and isofistularin-3 were evaluated using HPLC according to the geographic location of its habitat, significant variation was observed at the largest (Canary Islands and Mediterranean, over 2500 km) and the smallest (two sites less than 500 m apart) geographic scales. Another study evaluating the variability in metabolites of *Stylissa massa* came to a similar conclusion [23]. The concentrations of the bromopyrrole alkaloids—e.g., hymenidine analogs, sceptrin, and oroidin—were measured using LC-MS analysis. Concentrations varied geographically across the Pacific basin, with American Samoa and Pohnpei exhibiting the greatest differences, and Guam and Saipan being the most similar to each other.

A very recent metabolomics study revealed the relationship between sponge metabolites and the geographic location. Total 139 specimens of *Xestospongia* spp. were collected in four locations—Martinique, Curacao, Taiwan, and Tanzania—and the extracts were analyzed by ^1^H NMR and LC-MS, followed by statistical analysis (OPLS-DA); the collected samples were clearly grouped according to their location [24].

Experimental evidence supports this geographical trend of the sponge metabolites, although it is scattered throughout the literature. This review aims to compile this information and determine variations in metabolites according to geographical location using the genus *Petrosia*. *Petrosia* is one of the four genera (*Acanthostrongylophora*, *Neopetrosia*, *Petrosia*, *Xestospongia*) of the family *Petrosiidae* belonging to the order *Haplosclerida,* which is known as the most prolific source of secondary metabolites among sponges [12]. As of 2020, *Petrosia* genus includes 122 species belonging to two subgenera of *Petrosia* and *Strongylophora*, according to the Word Register of Marine Species (WoRMS). They are widely distributed throughout tropical and temperate waters, from intertidal zones to deep waters.

This review collects and compares information from peer-reviewed articles on secondary metabolites isolated from the sponges of the genus *Petrosia*. The articles were retrieved from the following databases: PubMed, Chemical Abstracts^®^, ISI Web of Knowledge, Google Scholar. Based on the assumption that the latitude of sponge habitat plays an important role in the metabolite, the regions were divided into polar (above the Arctic Circle (66.5° N) and below the Antarctic Circle (66.5° S)), temperate (between the Tropic of Cancer (23.5° N) and the Arctic Circle and between the Tropic of Capricorn (23.5° S) and the Antarctic Circle), and tropical area (between the Tropic of Cancer and the Tropic of Capricorn).

## 2. *Petrosia* Metabolites

### 2.1. Polyacetylenes

#### 2.1.1. Polyacetylenes Isolated from Temperate *Petrosia* Sponges

Polyacetylenes are characteristic metabolites of *Petrosia* sponges inhabiting temperate regions above the Tropic of Cancer. Of the 38 publications regarding the secondary metabolites from temperate *Petrosia* sponges, 31 reported the isolation of polyacetylenes.

Mixtures of high-molecular weight polyacetylenes with 46–55 carbons were isolated from both the sponge *P. ficiformis* and its predator nudibranch *Peltodoris atromaculata* [25] in the Mediterranean Sea. These were the first reported polyacetylenes from *Petrosia* sponges, although some of the structural features of these—e.g., the aliphatic carbon chain length between the characteristic functional groups and stereochemistry—were not revealed. Five years later, the same research group isolated small amounts of two additional polyacetylenes, but their structural assignment remained incomplete [26]. Mixtures of five additional polyacetylenes up to 52 carbons long, isolated from *P. ficiformis* found in dark caves in the Mediterranean Sea, were also reported [27].

**Figure 1:**
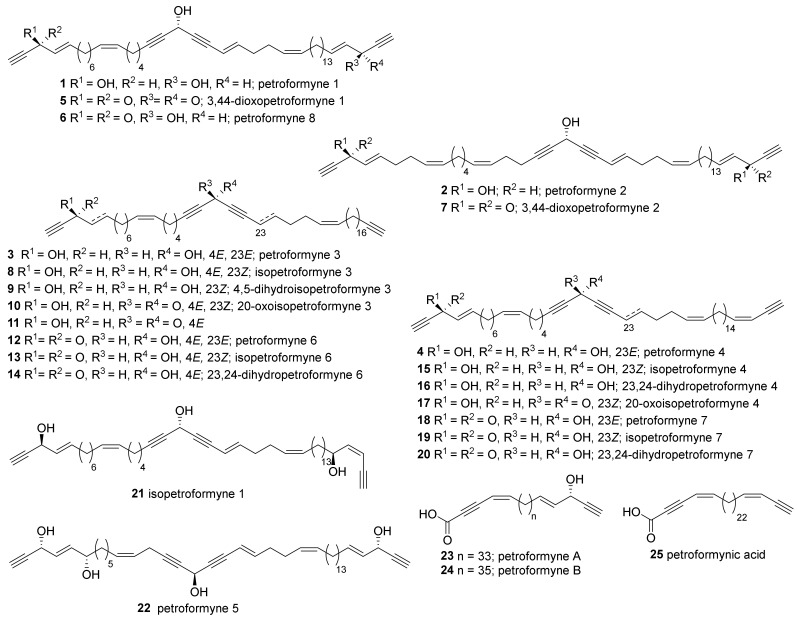
Polyacetylenes isolated from Mediterranean *Petrosia* sponges.

In 1989, polyacetylenes with two terminal 1-yn-3-ol-4-ene moieties and 46 total carbons isolated from Mediterranean *P. ficiformis* were completely identified and evaluated for their biological activity (Figure 1); the structures of petroformynes 1–4 (**1**–**4**) were elucidated, and it was shown that they inhibit sea urchin egg development and act as a potent toxin against *Artemia salina* [28]. The absolute stereochemistry of petroformynes was later elucidated by Mosher’s method [29]. The minor analogs contained in the same species were added through further research [30,31,32]; petroformynes, isopetroformynes, and various oxidized or isomerized analogs (**5**–**22**) were reported. Most of these analogs exhibited lethality against brine shrimp.

Polyacetylene carboxylic acids—petroformynes A, B (**23**, **24**), and petroformynic acid (**25**)—were also reported [32]. Compounds **23** and **24** showed high lethality in brine shrimp assay. Compounds **23** and **24** showed high lethality in brine shrimp assay.

**Figure 2:**
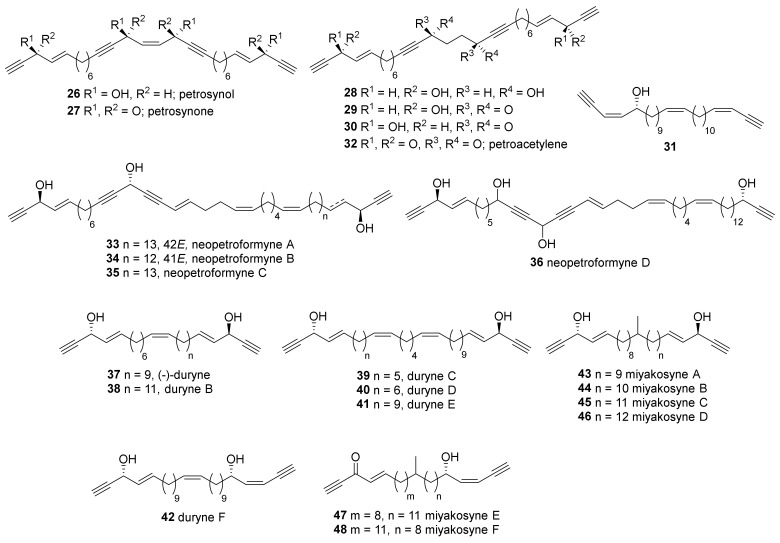
Polyacetylenic alcohols isolated from Japanese *Petrosia* sponges.

Isolation of polyacetylenes from *Petrosia* sp. inhabiting temperate regions of Japan was initiated from the discovery of the C30 polyacetylenes, petrosynol (**26**) and petrosynone (**27**) (Figure 2) [33,34]. In the case of petrosynol (**26**), the stereochemistry of hydroxymethine was revealed by measuring the Cotton effect of benzylated derivatives. This was the first report to completely elucidate the structure of polyacetylenes, including stereochemistry. Four more petrosynol derivatives (**28**–**31**) were reported by another research group [35]. These compounds inhibited the cell division of fertilized ascidian (*Styela partita*) eggs with IC_50_ values of 5.0–30.0 µg/mL and displayed toxicity in the brine shrimp lethality bioassay with LC_50_ values from 0.1–30.0 µg/mL. Petroacetylene (**32**), a tetraketone derivative of **29** and **30**, was also reported by a different research group [36].

The petroformyne analogs were also isolated from a Japanese *Petrosia* sponge; petroformynes 1 and 4 (**1**, **4**) [37], in addition to the novel derivatives, neopetroformynes (**33**–**36**), were isolated [38]. The functional groups were identified based on NMR spectroscopy data including those from 2D NMR experiments such as COSY, HMBC, and TOCSY. The length of the alkyl chains was determined by analyzing FAB-MS/MS data and stereochemistry, except for that of neopetroformyne D (**36**), was revealed by the modified Mosher method. In this report, the authors suggested that neopetroformyne A (**33**) and petroformyne 1 (**1**) might share the same structures, as their NMR data were indistinguishable. The structure of **1**, however, could not be reexamined by FAB-MS/MS due to a lack of remaining material. Neopetroformynes A–D (**33**–**36**) exhibited cytotoxicity against P388 murine leukemia cells with an IC_50_ values in ranging from 0.09 to 0.45 μg/mL.

The related analogs, named durynes (**37**–**42**), were reported by the same research group [39]. Compound **37** ((−)-duryne) was found to be an enantiomer of (+)-duryne, which has previously been isolated from the marine sponge *Cribrochalina dura* collected off the shore of Staniel Cay (24.2° N) in the Bahamas [40]. The taxonomy of *Cribrochalina dura* was later revised to be *Petrosia*, and the absolute configuration of each enantiomer was confirmed by the synthesis of both enantiomers [41].

Miyakosynes are also allyl propargyl alcohols or ketones like petroformyne derivatives, but they have a branched methyl group in the aliphatic chain in the middle. Six miyakosynes (A–F, **43**–**48**) were isolated from the sponge *Petrosia* sp. collected at Miyako sea knoll in Japan [42]. The locations of methyl branches were determined by tandem FAB-MS/MS analysis, and the stereochemistry was assigned using the modified Mosher’s method. Compounds **43**–**46** and a mixture of **47** and **48** exhibited cytotoxicity against HeLa cells with IC_50_ values of 0.10, 0.13, 0.04, 0.15, and 0.30 μg/mL.

**Figure 3:**
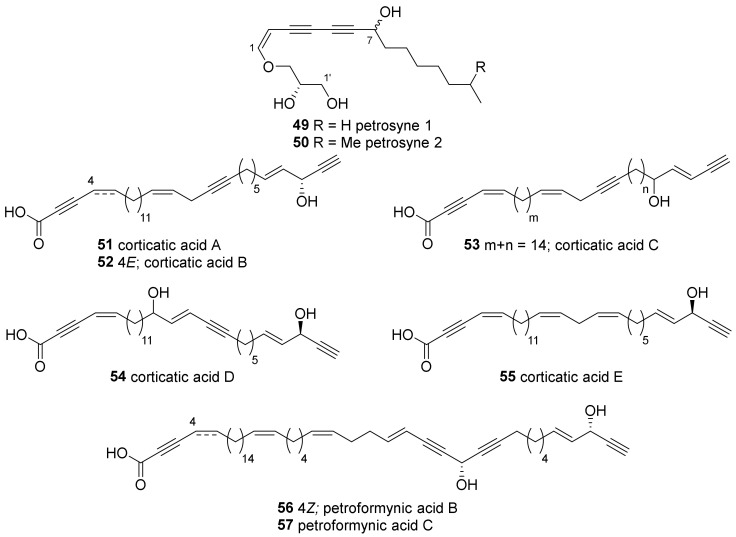
Polyacetylenic enol ether glyceride and carboxylate from Japanese *P.* sponges.

Petrosynes (**49**, **50**) are polyacetylenic enol ethers isolated from a *Petrosia* sp. collected near Ishigaki Island (Figure 3) [43]. Followed by the deduction of the plane structure by spectroscopic analysis, all possible stereoisomers of **49** and **50** were synthesized enantioselectively, which showed both to consist of mixtures of 7*R* and 7*S* diastereomers.

Polyacetylene carboxylic acids are another group of compounds isolated from Japanese *Petrosia* sponges. Five corticatic acids (**51**–**55**), which show antifungal activity against *Mortierella ramanniana* or *Candida albicans*, have been isolated from *P. corticata* [44,45]. Petroformynic acids B and C (**56**, **57**)—analogous to petroformynes 4 and 3 (**4**, **3**), respectively—were isolated along with petroformynes 1 and 4 (**1**, **4**) [37]. Compounds **56** and **57** inhibited the growth of P388 cells with an IC_50_ value of 0.4 μg/mL.

**Figure 4:**
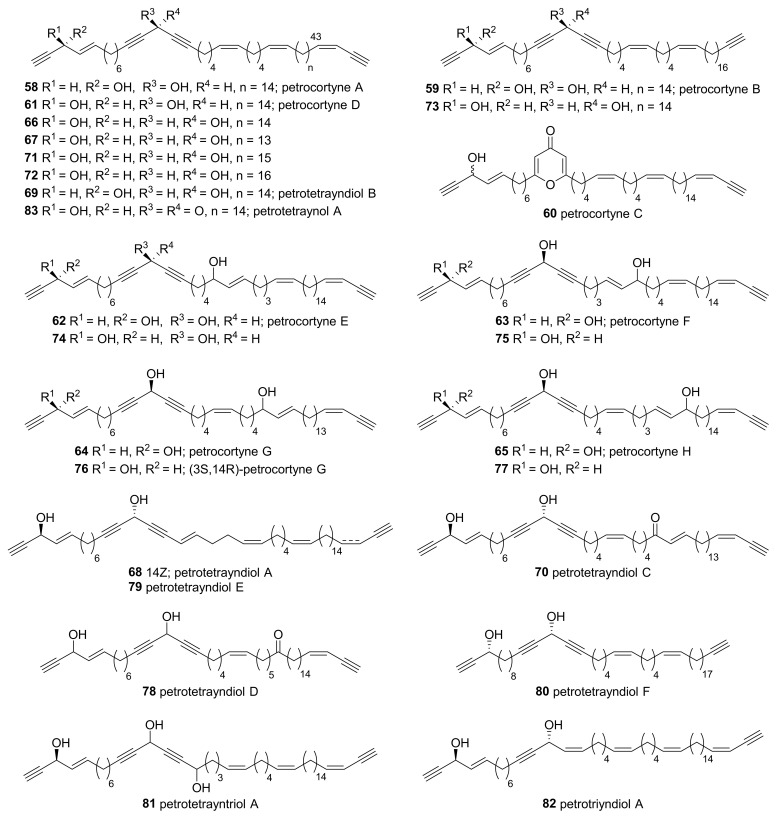
Petrocortyne derivatives isolated from Korean *Petrosia* sponges.

Polyacetylenes isolated from *Petrosia* inhabiting Korean water are mostly petroformyne 1 (**1**) and petrosynol (**26**) analogs, which have at least one allyl propargyl alcohol at the terminus (Figure 4). Petrocortynes A–H (**58**–**65**) were isolated from the extract of *Petrosia* sp. collected along offshore from Keomun Island, South Sea of Korea [46,47]. The structures of these compounds were elucidated by extensive NMR experiments, including COSY, HETCOR, HMQC, HMBC, and TOCSY, and the stereochemistry was revealed by a modified Mosher’s method. Petrocortyne C (**60**) possesses an unusual γ-pyrone ring, while the other analogs are linear C46 tetraacetylenes with a diacetylenic carbinol functionality in the middle. Petrocortynes A–C (**58**–**60**) showed significant brine-shrimp lethality and RNA-cleaving activity and petrocortynes D–H (**61**–**65**) exhibited moderate to minimal cytotoxicity against a human leukemia cell line (K562) with LC_50_ values of 7.0–45.0 µM.

Derivatives of the above mentioned petrocortynes appear in the several reports regarding Korean *Petrosia* sponges. Isolation of (3*S*, 14*S*)-petrocortyne A (**66**), nor-(3*S*,14*S*)-petrocortyne A (**67**), and petrotetrayndiols A–C (**68**–**70**) from the *Petrosia* inhabiting the same area was later reported [48,49]. Additional petrocortynes (**71**–**77**) and petrotetrayndiol derivatives (**78**–**83**) were isolated and reported by another research group [50,51]. Most of these compounds showed high cytotoxicity against several human solid tumor cell lines.

**Figure 5:**
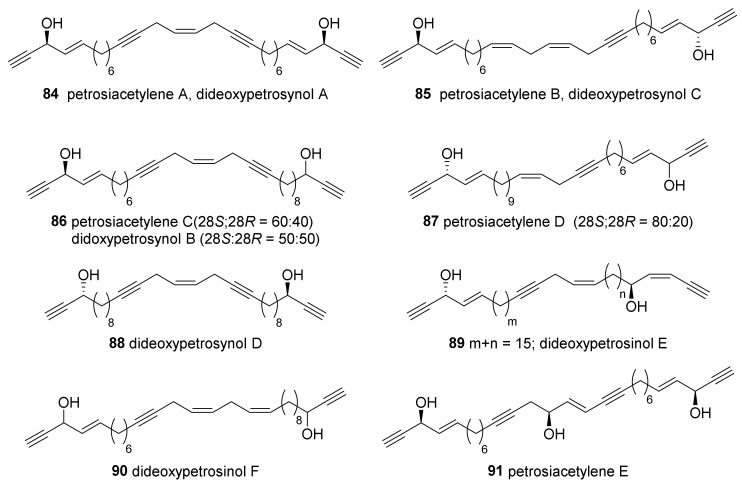
Petrosiacetylenes isolated from Korean *Petrosia* sponges.

Seo et al. reported the isolation of C30 polyacetylenes analogous to petrosynol (**26**) from the above mentioned *Petrosia* sp. collected near Keomun Island (Figure 5) and named these petrosiacetylenes A–D (**84**–**87**) [46]. Compounds **86** and **87** are mixtures of diastereomers at C-28. In the same year, another research group reported the compounds of the same type (dideoxypetrosynols A–D) along with petrosynol (**26**) and duryne (**37**) [52]. Dideoxypetrosynols A–C later turned out to be the same compounds as petrosiacetylenes A (**84**), C (**86**), and B (**85**), respectively, although the diasteromeric ratio of petrosiacetylene C (28*S*:28*R* = 60:40) and dideoxypetrosynol B (28*S*:28*R* = 50:50) were slightly different. Dideoxypetrosynol D (**88**) was also a mixture of 28*S* and 28*R* diastereomers. Additional dideoxypetrosynols (**89**, **90**) were reported, although the positions of multiple bonds and the stereochemistry have not been revealed [48]. Petrosiacetylene E (**91**) was isolated and its structure was completely elucidated [53]. Compared to analogous petrosiacetylenes A (**84**) and C (**86**), compound **91** showed three times higher cytotoxicity against a panel of human cancer cell lines, which emphasized the importance of the hydroxygroup at C-15.

**Figure 6:**
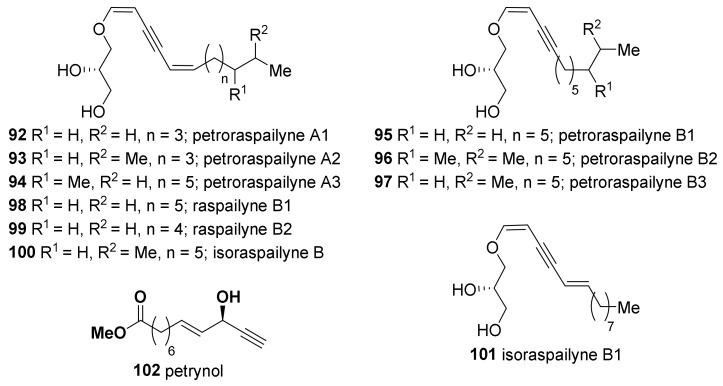
Polyacetylenic enol ether glycerides and carboxylates isolated from Korean *Petrosia* sponges.

Novel enol ether glycerides (**92**–**97**) were isolated from the above mentioned *Petrosia* sponge collected near Keomun Island, along with analogous raspailynes and isoraspailynes (**98**–**101**) which have previously been isolated from the sponges *Raspailia pumila* and *Raspailia ramosa* (Figure 6) [54,55]. In the measurement of cytotoxicity against a human leukemia cell line (K562), the yne-diene derivatives (**92**–**94**) exhibited weak activity (LC_50_ 9.2, 57.0, and 29.0 μg/mL, respectively), while the derivatives **95**–**97**, possessing the yne-ene moiety, were not active. The carboxylate (**102**), which has an acetylenic alcohol moiety and a linear C12 carbon chain, was also reported in the same paper. This is reminiscent of petroformynes (**23**, **24**) and petroformynic acid (**25**), which were isolated from Mediterranean *P. ficiformis*.

#### 2.1.2. Polyacetylenes Isolated from Tropical *Petrosia* Sponges

Several polyacetylenes have been isolated from tropical *Petrosia* sponges, although not as many as those from their counterparts in temperate regions. These compounds can be categorized into two groups: one comprised of compounds bearing a diynol group and the other comprised of enyne carboxylates.

**Figure 7:**
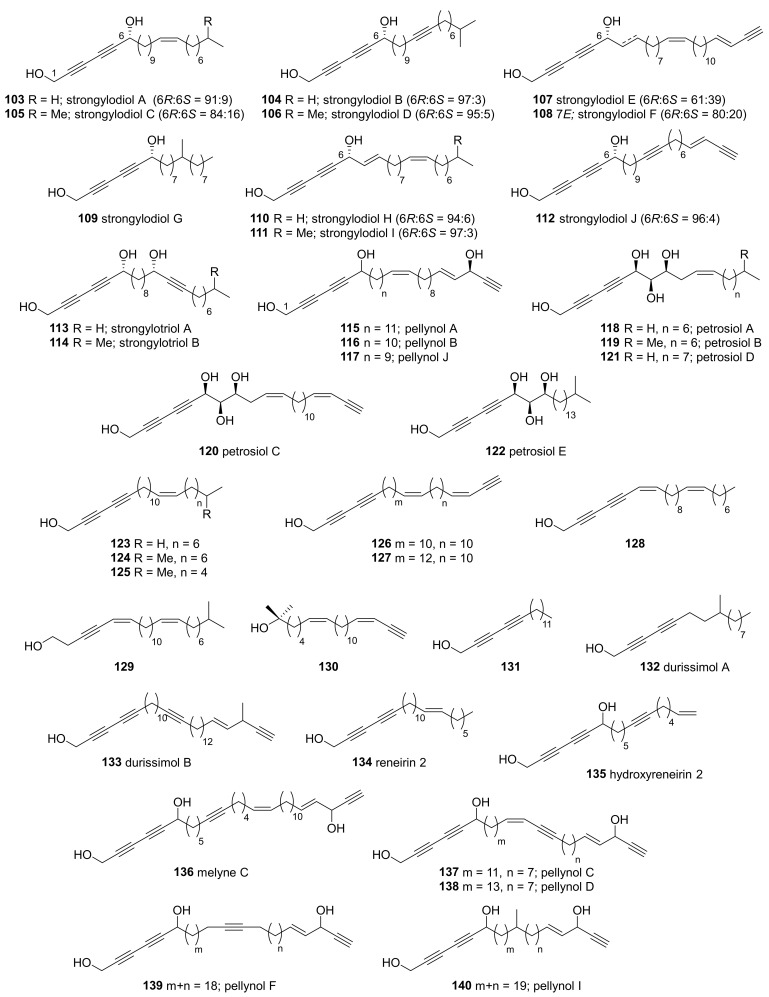
2,4-Diyn-1-ols isolated from tropical *Petrosia* sponges.

Alcohols with 2,4-diyne (the first group) were isolated from the Okinawan sponges of *Petrosia* genus (Figure 7). Strongylodiols A–J (**103**–**112**) were isolated from an Okinawan *Strongylophora* sponge that was later revised as *Petrosia* [56,57]. These compounds were isolated as mixtures of enantiomers at C-6, which were resolved by modified Mosher’s method.

Analogous triols, strongylotriols (**113**, **114**), and pellynols (**115**–**117**) were also isolated from an Okinawan *Petrosia* sponge [58]. The strongylotriols were found to be optically pure, whereas the pellynols were diasteromeric mixtures at C-6. The strongylotriols and pellynols exhibited different levels of cytotoxicity against HeLa and K562 cell lines; strongylotriols A and B (**113**, **114**) exhibited IC_50_ values of 11.3–18.1 µM, and pellynols A, B and J (**115**–**117**) exhibited IC_50_ values of 0.5–0.6 µM.

The tetraol derivatives, petrosiols (**118**–**122**), were isolated along with strongylodiols C and D (**105**, **106**) from an extract of the Okinawan sponge *P. strongylata* [59]. The stereochemistry of the petrosiols was determined by derivatizing petrosiol C (**120**); **120** was hydrogenated using a Pt catalyst to provide the fully saturated derivative, then the stereochemistry of the product was assigned by comparing its ^1^H NMR to those of the four stereoisomers of 1,2,6,7-decanetetraol provided in Kishi’s Universal NMR databases [60]. Petrosiols (**118**–**122**) induced nerve growth factor-like neuronal differentiation of PC12 cells, which suggested the importance of the rare 2,4-diyne-1,6,7,8-tetraol fragment in neurotrophic activity.

The same type of acetylenic alcohols (**123**–**131**) were additionally isolated from an Okinawan *Petrosia* sp. [61]. In the regarding report, it is described that the position of the central double bonds in compounds was revealed by chemical transformation—the oxidative cleavage of the olefin, followed by the transformation of resulting aldehyde into the corresponding 2,4-dinitrophenylhydrazone. The analysis of the HRMS and ^13^C-NMR spectral data of each resulting hydrazones clarified the position of the central double bonds in **123**–**131**.

Additional 2,4-diyn-1-ols were also isolated from *Petrosia* sponges growing in other tropical areas. Durissimols A and B (**132**–**133**) were isolated from *P. durissima* collected in Taiwan, along with the previously reported derivatives (**37**, **134**–**136**) isolated from *Reniera fulva* [62]. Among the compounds isolated, duryne (**37**) and durissimol B (**133**) exhibited potent cytotoxicity against human gastric cancer cells (NUGC). Pellynol derivatives (**115**, **137**–**140**) were isolated from a *Petrosia* sp. collected in American Samoa [63].

**Figure 8:**
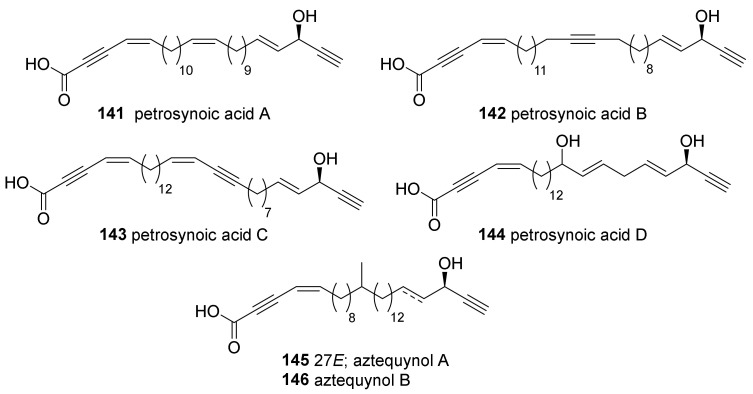
Enyne carboxylates isolated from tropical *Petrosia* sponges.

Enyne carboxylates (**141**–**144**), comprising the second group of petrosiacetylenes in tropical *Petrosia*, were isolated from the sponge mentioned directly above (Figure 8) [63].

In the measurement of cytotoxicity against three human cancer cell lines (A2058, H522-T1, H460) and one non-proliferating human fibroblast (IMR-90), all nine compounds (**115**, **137**–**144**) were active against human cancer cell lines with IC_50_ values ranging of 0.4–2.7 µM; however, all compounds were also similarly potent against the IMR-90 cells.

In an earlier study on tropical *Petrosia* sp., aztèquynols A and B (**145**, **146**) were isolated from the cytotoxic extract of a *Petrosia* sp. collected in New Caledonia [64]. The structures were assigned by NMR spectroscopy, FAB MS/MS, and chemical transformation; however, neither of the compounds accounted for the cytotoxicity of the sponge extract against KB tumor cell lines (less than 20% cytotoxicity at 10 μg/mL).

### 2.2. Terpenoids and Related Compounds

#### 2.2.1. Sterols Isolated from Tropical *Petrosia* Sponges

Unique sterols with an extended side chain have been isolated from *Petrosia* sponges. Sterols were isolated from tropical *Petrosia* more frequently. There are 12 papers reporting the isolation of 40 sterols from tropical *Petrosia* sp., while four papers regarding five sterols from temperate counterparts.

**Figure 9:**
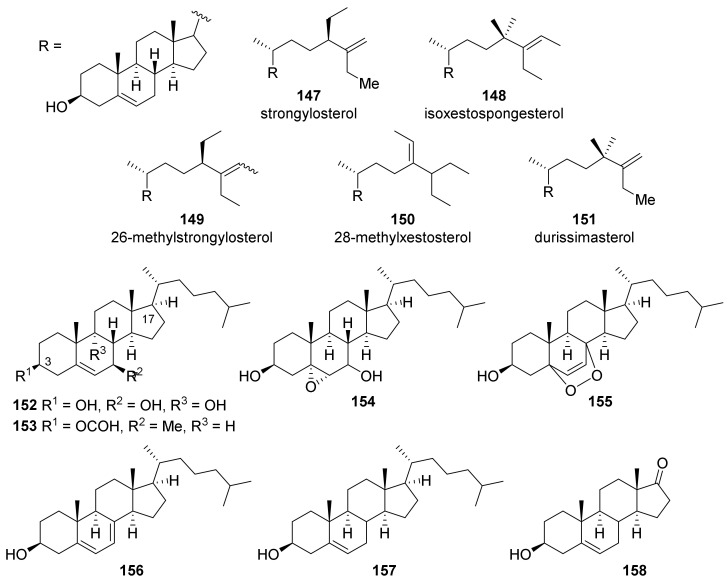
Sterols isolated from tropical *Petrosia* sponges.

The first *Petrosia*-derived sterol was isolated from Indo-Pacific *S. durissima* [65] (Figure 9). The structure of this sterol (**147**), named strongylosterol, was completely resolved later by chemical synthesis [66]. Subsequently, additional minor sterols (**148**–**151**) were isolated from the extract of the same species, and the structures were elucidated by comparing spectroscopic data with those obtained for synthesized derivatives [67,68].

Oxygenated cholestane derivatives were isolated from a *Petrosia* sp. collected off the Saudi Arabian Red Sea coast (21° N) [69]. The compounds in this series—containing a tetracyclic skeleton of 3,7,9-trihydroxycholastane (**152**)—are isolated in the form of oxidized derivatives, i.e., formate (**153**), epoxide (**154**), peroxide (**155**), and diene (**156**). Compounds with the reduced C-7 (**157**) or a carbonyl group at C-17 (**158**) were also isolated. For these compounds, cytotoxicity against human hepatocellular carcinoma (HepG2) and human breast adenocarcinoma (MCF7) was measured; however, none of the compounds showed a meaningful level of activity.

**Figure 10:**
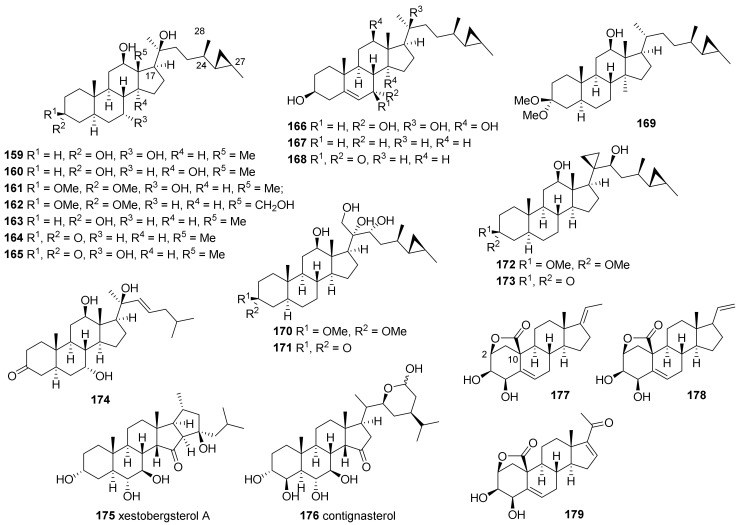
Polyhydroxysterols isolated from tropical *Petrosia* sponges.

The C29-steroids with a cyclopropane ring at C-25–C-27 (**159**–**174**) were isolated from the Thai sponge of *Petrosia* genus (Figure 10) [70]. Based on the assumption that compounds with 3-dimethyl ketal functionality (**161**, **162**, **169**, **170**, **172**) might have been artificially formed during isolation and purification, compounds with a carbonyl group at C-3 (**165**, **173**) were subjected to conditions similar to those during isolation and purification. No change in the thin layer chromatography analyses was observed, even after one month, suggesting that isolated dimethyl ketal derivatives occur naturally. Compounds—except for **162**, **167** and **169**—were evaluated for cytotoxicity against six human cancer cell lines and a normal cell line. Compound **173** was the most potent, with IC_50_ values of 7.1 and 6.1 μΜ against HepG2 and HeLa cell lines, respectively. All the other compounds exhibited minimal to weak cytotoxicity, with IC_50_ values in the range of 11.2–103.5 μΜ.

Xetobergsterol A (**175**) and contignasterol (**176**), which contain a highly oxygenated cyclic skeleton and a ketone moiety at C-15, were isolated from *P.* cf. *contignata* and *P. contignata* Thiele, respectively [71,72]. Both compounds contain a 14β hydrogen, which is very rare in naturally occurring steroids, although steroids with a 14β-hydroxyl functionality (i.e., digitoxin) are well-known in nature.

Three pregnane derivatives (**177**–**179**) bearing the unique 2,10-carbolactone moieties were isolated from *Strongylophora* (*Petrosia*) sp. collected at Puako, Hawaii [73]. The structures were assigned based on spectroscopic data and confirmed by X-ray crystallography of **177**. Compounds **178** showed marginal cytotoxicity against human epidermoid carcinoma (KB) and human colorectal adenocarcinoma (LoVo) cell lines with MIC values of 1.0 and 5.0 μg/mL, respectively, while compound **177** and **179** showed no activity.

**Figure 11:**
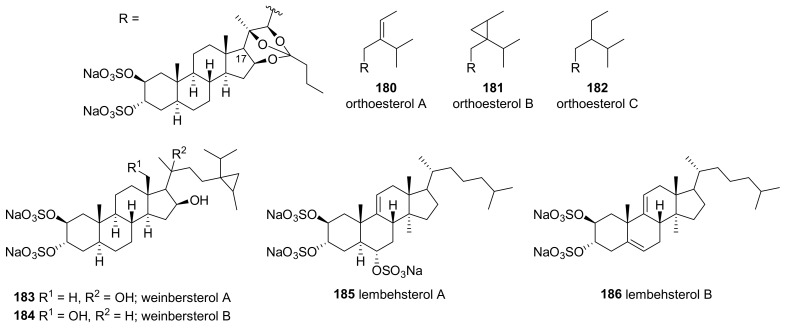
Sulfated sterols isolated from tropical *Petrosia* sponges.

Sulfated sterols with an ortho ester (orthoesterols A–C, **180**–**182**) were isolated from the extract of *P. weinbergi* collected in the Bahamas (Figure 11) [74]. The compounds were isolated through the bioassay-guided separation of the extract, which showed activity against feline leukemia virus (FELV), mouse influenza virus (PR8), and mouse coronavirus (A59) and all three (**180**–**182**) showed activities against FELV and PR8.

Weinbesterol A and B (**183**, **184**), sulfated tetrahydroxysteroids with a cyclopropane-containing side chain, were isolated from the *P. weinbergi* collected in the Bahamas [75]. The structures were resolved using extensive NMR experiments, including 2D INADEQUATE, HMBC, HETCOR, COSY, and COLOC. Both were active against FELV (EC_50_ = 4.0 and 5.2 μg/mL, respectively), and **183** was active against HIV (EC_50_ = 1.0 μg/mL).

Lembehsterols A and B (**185**, **186**) were isolated from *P. strongylata* collected at Lembeh Island, Indonesia [76]. Both showed inhibitory activity against thymidine phosphorylase (IC_50_ = 41.0 and 45.0 μM, respectively) which is related to angiogenesis in solid tumors. Desulfated derivatives obtained by the treatment of **185** with acid showed no inhibition at 230.0 μM, suggesting the importance of the sulfate group for the activity against thymidine phosphorylase.

#### 2.2.2. Sterols Isolated from Temperate *Petrosia* Sponges

**Figure 12:**
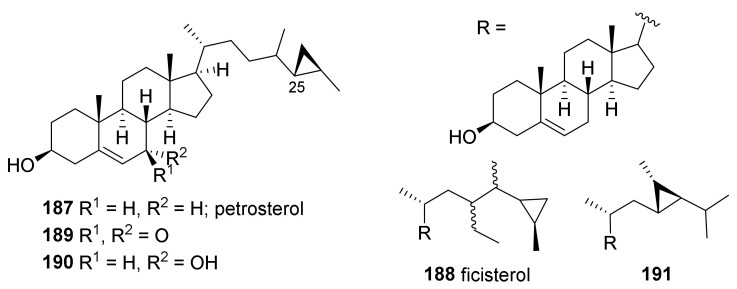
Sterols isolated from temperate *Petrosia* sponges.

Sterols have rarely been isolated from *Petrosia* sponges in temperate regions. Only two species, *P. ficiformis* and *S. corticata*, were found to contain sterol compounds. All the isolated sterols from both contain cyclopropane in the branch at C-17 (Figure 12). Petrosterol (**187**), a steroid with a cyclopropane ring at C-25 and C-26, was isolated from *P. ficiformis* collected in the Bay of Naples, and its structure was confirmed by the X-ray crystallography of a synthesized p-bromobenzoate derivative [77,78]. Subsequent studies tracing the minor component of the same species resulted in the isolation of ficisterol (**188**) [79]. 7-Oxo and 7-hydroxy derivatives of petrosterols (**189**, **190**) were isolated from *S. corticata* collected off the coast of Tokushima (33° N) [80] along with petrosterol (**187**) and dihydrocalysterol (**191**). Compound **191** has been isolated from the sponge *Calyx niceaensis*, previously [81]. The biological activity of the petrosterol analogs (**187**–**191**) have not been reported yet.

#### 2.2.3. Meroterpeonids Isolated from Tropical *Petrosia* Sponges

Meroterpenoids are a group of compounds that are partially derived from the terpenoid biosynthetic pathway. In the marine environment, meroterpenoids have been isolated most frequently from brown algae and microorganisms, but another important source is marine invertebrates, such as sponges and tunicates [82]. All the meroterpenoids isolated from *Petrosia* sponges are from sponges collected in tropical areas.

**Figure 13:**
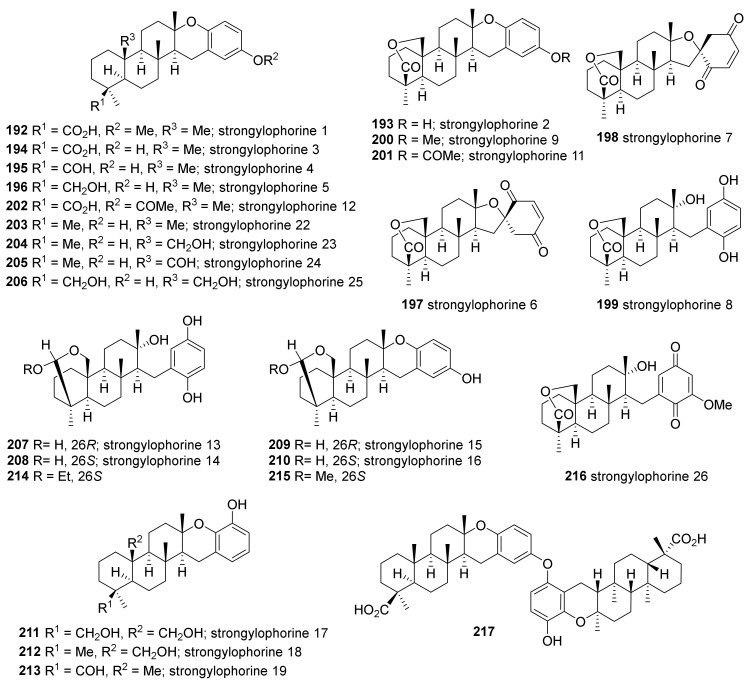
Strongylophorine derivatives isolated from tropical *Petrosia* sponges.

Strongylophorines, which are meroditerpenoids with a quinol or a quinone moiety, are the most abundant metroditerpenoids in *Petrosia* sponges (Figure 13). Initially, strongylophorines 1–3 (**192**–**194**) were isolated from *S. durissima* collected in Papua New Guinea and identified based on their spectral properties, chemical synthesis, and X-ray diffraction analysis [83]. Five additional analogs, strongylophorines 4–8 (**195**–**199**), were isolated from the same species collected in the Philippines [84]. These compounds (**192**–**199**) exhibited mild antimicrobial activity against *Bacillus subtilis* and *Staphylococcus aureus*, with the two quinones (**197**, **198**) showing the greatest inhibition. Strongylophorines 9, 11 and 12 (**200**–**202**) were isolated from the same species inhabiting Taiwanese water, along with strongylophorine 1–3 (**192**–**194**), and 5 (**196**) [62,85].

Eighteen strongylophorines (**192**–**195**, **199**, **203**–**215**) were isolated from Okinawan *Petrosia* sponges. From *P. corticata*, strongylophorines 1–4 (**192**–**195**), 8 (**199**), 15–16 (**209**, **210**), and 22–24 (**203**–**205**) were isolated and structures were identified by spectroscopic analysis and chemical transformation [86]. Among these compounds, strongylopohrine 22 (**203**) showed moderate cytotoxicity against HeLa cells with an IC_50_ value of 26.6 μΜ. Eleven derivatives—strongylophorines 2–4 (**193**–**195**), 8 (**199**), 13–19 (**207**–**213**)—were isolated from *S. strongylata* [87]. Except for strongylophorine 4 (**195**), all compounds inhibited the maturation of starfish oocytes with IC_50_ values of 1.1–37.6 μM. The same species inhabiting in the same area were revisited after 10 years, which resulted in the isolation of additional analogs, **214** and **215**, the acetal derivatives of strongylophorines [88]. In this study, the inhibitory activity of all the obtained strongylophorines (**193**, **194**, **199**, **209**, **211**, **214**, **215**) was measured against protein tyrosine phosphatase 1B (PTP1B), which plays a key role in the negative regulation of the insulin and leptin signaling pathways. Most of the compounds showed activity with **214** and **215** being the most potent (IC_50_ 8.7 and 8.5 μM, respectively).

Strongylophorines, which are the same as the above mentioned compounds from Okinawan *Petrosia*, were isolated from *P. corticata* collected in North Sulawesi, Indonesia [89]. In this study, the inhibitory activities of the isolated compounds (**193**–**195**, **199**, **203**, **207**, **208**, **214**, **215**) were tested against the chymotrypsin-like activity of proteasome. The mixture of diasteromeric hemiacetals (strongylophorines 13, 14; **207**, **208**) were the most potent (IC_50_ 2.1 μM), and the acetal compounds (**214**, **215**) were slightly less potent (IC_50_ 9.3 and 6.6 μM, respectively), while the analogous lactone (**193**) showed no activity at 100 μM. Compounds **194**, **195**, and **203**—bearing a carboxylate, an aldehyde, and a methyl group at C-4, respectively—showed the activities decreased in the same order (IC_50_ 9.5, 19.0, 100.0 μM, respectively).

Strongylophorine 26 (**216**) was isolated from the *P. corticata* collected in Papua New Guinea, along with strongylophorine 8 (**199**) [90]. Compound **216** exhibited an IC_50_ value of approximately 1.0 μg/mL in the anti-invasion assay using MDA-231 cells, while **199** showed considerably less potency (IC_50_, ~7.0 μg/mL).

A dimeric strongylophorine (**217**), composed of two equivalents of strongylophorine 3 (**194**), was isolated from a Philippine marine sponge of the genus *Strongylophora* (*Petrosia*), along with strongylophorines 2–4 (**193**–**195**) [91]. In this study, compounds **194** and **195** showed marginal activity against *Micrococcus luteus* and *Salmonella typhi*, respectively. Compound **194** was also active against the phytopathogenic fungus *Cladosporium cucumerinum* and the neonate larvae of the polyphagous insect pest *Spodoptera littoralis* (EC_50_ 69.0 μg/mL). Compound **217** was the most active in the brine shrimp lethality assay, with an LC_50_ value of 10.5 g/mL.

**Figure 14:**
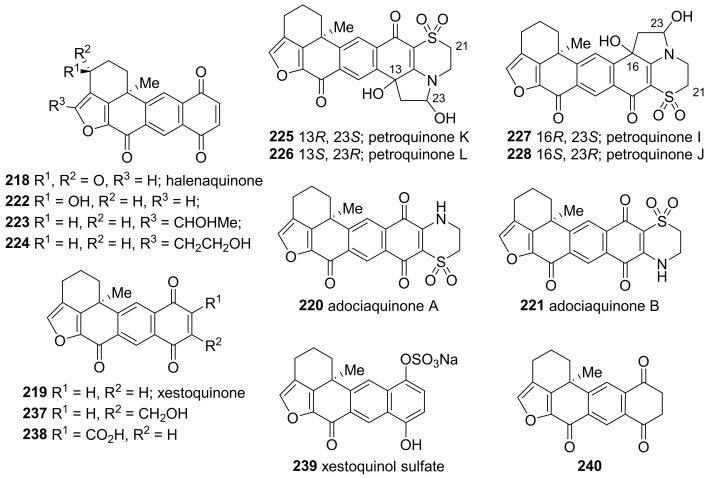
Monomeric halenaquinone derivatives isolated from tropical *Petrosia* sponges.

Pentacyclic quinones with a furan moiety make up another group of meroditerpenoids from *Petrosia* sponges (Figure 14). Halenaquinone (**218**), xestoquinone (**219**), and adociaquinones (**220**, **221**) are representatives of petacyclic quinones, originally isolated from sponges of the genus *Xestospongia* [92,93] or *Adocia* [94] (Schmitz and Bloor 1988).

**Figure 15:**
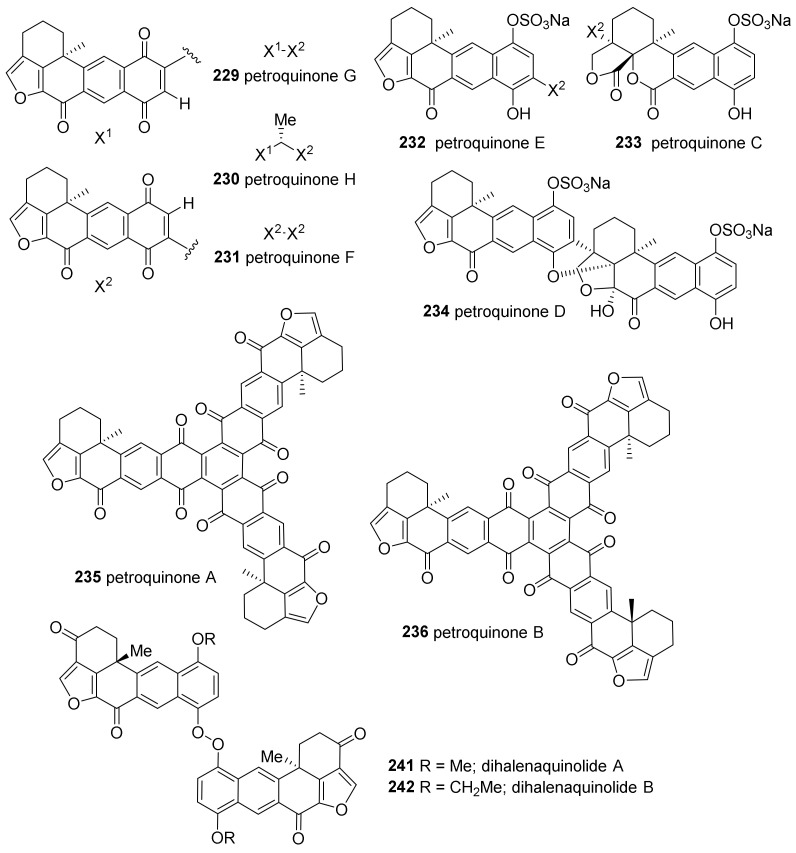
Dimeric and trimeric halenquinone derivatives isolated from tropical *Petrosia* sponges.

A series of halenaquinone derivatives—including eight monomers (**218**, **222**–**228**), six dimers (**229**–**234**), and two trimers (**235**, **236**)—were isolated from *P. alfiani* collected in Indonesia (Figure 14 and Figure 15) [95]. The structures were identified based on their NMR spectra, ECD spectra, and DFT calculations. All compounds, except for **222** and **223**, were evaluated for their inhibitory activity against ubiquitin-specific protease 7 (USP7), an enzyme related to the tumor suppression. All tested compounds exhibited potent activities with IC_50_ values of 0.1–2.0 μM.

The bioassay-guided separation of the extract of *P. alfiani* collected off the coast of Malaysia resulted in the isolation of xestoquinone derivatives (**219**–**221**, **237**–**240**) (Figure 14) [96]. The extract selectively inhibited iron chelator-induced hypoxia-inducible factor-1 (HIF-1) in the human breast tumor (T47D) cell-based reporter assay, and adociaquinones A (**220**) and B (**221**) were proven to be the active pharmaceutical ingredients (IC_50_ 0.2 μM against HIF-1). Mechanistic studies revealed that both compounds promote oxygen consumption without affecting mitochondrial membrane potential. In addition, four compounds (**219**–**221**, **237**) including adociaquinones (**220**, **221**) exhibited potent cytotoxicity (IC_50_, 2.6–6.5 μΜ) against two human breast cancer cell lines, T47D and MDA-MB-231, in a cell viability assay.

The dimers of halenaquinol (**241**, **242**) connected through peroxide linkage have been isolated from *P. elastica* collected in Taiwan (Figure 15) [97]. In this study, halenaquinone (**218**) and halenaquinol could be obtained by degrading **241**. The natural dimers (**241**, **242**) exhibited higher growth inhibitory activity compared to the synthesized monomeric counterparts (**218**, halenaquinol) against human hepatoma cancer cells (Hep3B) at 10.0 μg/mL (5% versus 47% and 20%, respectively).

**Figure 16:**
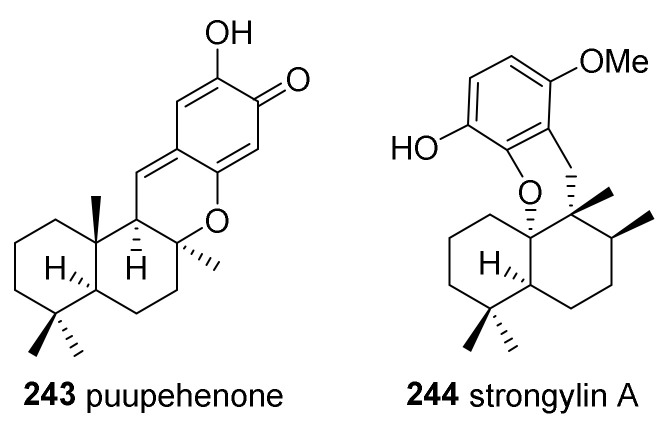
Merosesquiterpenoids isolated from *S. hartmani.*

A sesquiterpene-methylene quinone, puupehenone (**243**), was isolated from *S. hartmani* van Soest collected in the Bahamas (Figure 16) [98]. This compound was originally reported as a metabolite from a sponge of the genus *Hetronema* [99]. A sesquiterpene hydroquinone, strongylin A (**244**), was isolated from the same species later [100]. Strongylin A exhibited cytotoxicity against a murine leukemia cell line (P388) and influenza virus (PR8), with IC_50_ values of 13.0 and 6.5 μg/mL, respectively.

#### 2.2.4. Saponins Isolated from Indonesian *Petrosia* sp.

**Figure 17:**
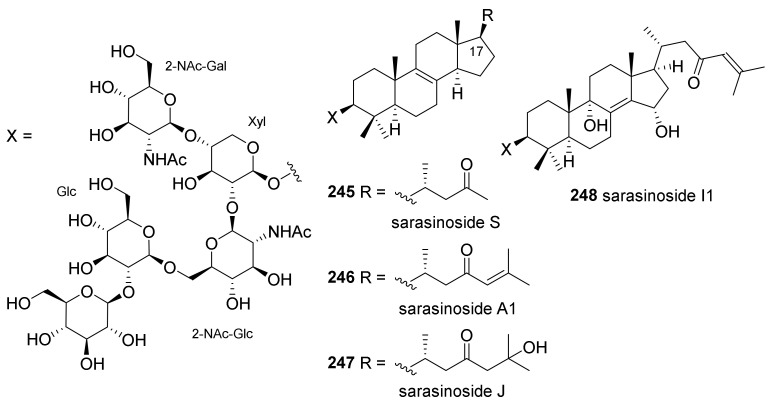
Saponins isolated from an Indonesian *Petrosia* sp.

Lanostane-type triterpene oligoglycosides, sarasinosides (**245**–**248**), were isolated from the Indonesian *Petrosia* sponge (Figure 17) [101]. Sarasinosides were originally isolated from marine sponges of the genera *Asteropus*, *Melophlus*, and *Lipastrotethya* [102,103,104], which belong to the same subclass (*Heteroscleromorpha*) as *Petrosia*. Compound **245**, named sarasinoside S, was isolated for the first time and its structure was elucidated based on its spectroscopic data. None of these isolated sarasinosides exhibited cytotoxicity against human solid cancer cell lines, Huh-7 and A549, although some sarasinoside derivatives have been reported to exhibit weak cytotoxicity against several cancer cell lines.

### 2.3. Alkaloids and Peptides

#### 2.3.1. Alkaloids Isolated from Tropical *Petrosia* Sponges

**Figure 18:**
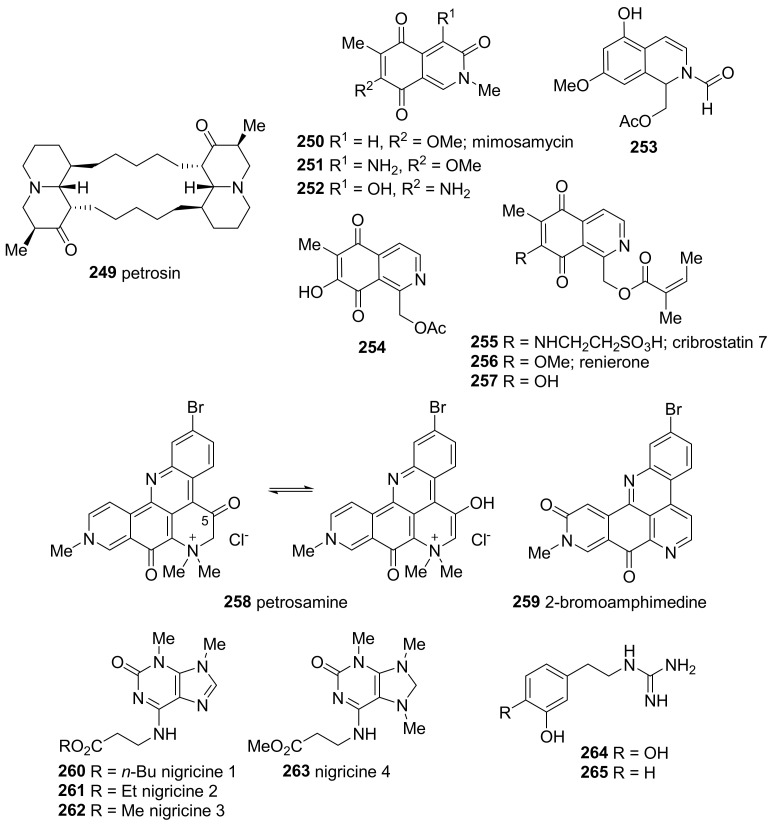
Alkaloids isolated from tropical *Petrosia* sponges.

Several alkaloids have been isolated from *Petrosia* sponges inhabiting tropical regions, whereas no alkaloids from the temperate *Petrosia* have been reported. Petrosin (**249**), a bisquinolizidine isolated from *P. seriata* collected in Papua New Guinea, is the first reported *Petrosia*-derived alkaloid (Figure 18) [105].

Isoquinoline alkaloids have been isolated from *Petrosia* sponges. Mimosamycin (**250**) and related derivatives (**251**–**254**) were isolated from *Petrosia* sp. and *P. similis* collected in India [106,107]. Isoquinoline quinones analogous to **254** (**255**–**257**) were isolated from two Philippine *Petrosia* sponges [108]. These compounds exhibited low cytotoxicity with an IC_50_ ranging from 24.0 to 45.0 μg/mL against the HCT116 human colon carcinoma cell line.

Petrosamine (**258**), a pyridoacridine alkaloid, was isolated from the *Petrosia* sp. collected in Belize [109]; it was confirmed to exist as a keto form by X-ray crystallography. However, a signal corresponding to that of a C-5 carbonyl group (δ_C_ 161 ppm) was detected, which suggests that it takes an enol form in solution. 2-Bromoamphimedine (**259**) was isolated later from a Thai marine sponge along with **258** [110]. Compound **258** showed strong acetylcholinesterase inhibitory activity approximately six times higher than that of galantamine that used as a reference (IC_50_ 0.09 vs. 0.59 μM), while **259** was totally inactive.

Purine derivatives (**260**–**263**) conjugated with a β-amino acid were isolated from *P. nigricans* collected in Indonesia, and named nigricines [111]. None of these compounds showed cytotoxicity against the murine lymphoma cell line (L5178Y) at 10.0 μg/mL, and no other activities could be measured due to the limited quantities obtained.

Phenethylguanidine alkaloids isolated from Indo-Pacific marine sponge *P.* cf. *contignata*, (**264**, **265**) are close derivatives of tubastrine [71]. Tubastrine was originally reported as a metabolite of the soft coral *Tubastrea aurea*, and compound **264** has previously been synthesized by the hydrogenation of tubastrine [112].

#### 2.3.2. Peptides Isolated from a Korean *Petrosia* sp.

**Figure 19:**
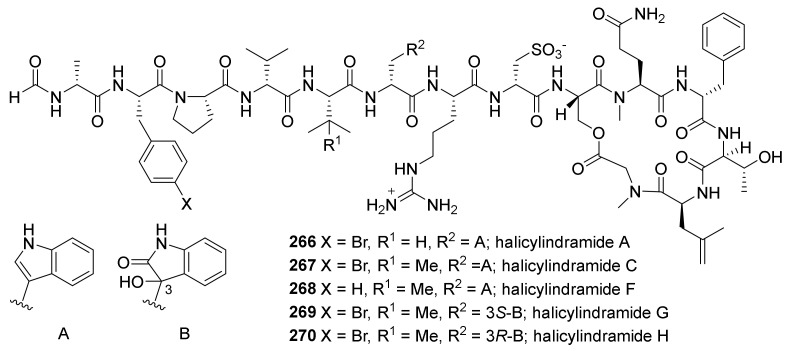
Depsipeptides isolated from a Korean *Petrosia* sp.

So far, only one article has reported the isolation of peptides from *Petrosia* sponge. This article described the isolation of five halicylindramides (**266**–**270**) from the *Petrosia* sp. collected in Korea (Figure 19) [113]. The structures of newly reported compounds (**268**–**270**) were elucidated based on their spectroscopic and mass data, and the stereochemistry was assigned by Marfey’s method and ECD spectroscopy. The article points out that the producers of these compounds might be sponge-associated cyanobacteria, as all of the previously reported halicylindramides were discovered from the sponge *Halichondria cylindrata*. Compounds **266**–**268**, bearing indole moiety at R_2_ position, showed antagonistic activity against human farnesoid X receptor (hFXR) with IC_50_ values of 6.0, 0.5, and 5.0 μM, respectively, while **269** and **270** were inactive even at 100.0 μM.

### 2.4. Fatty Acid Derivatives

**Figure 20:**
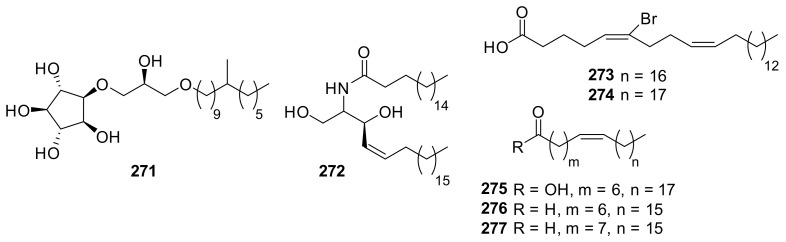
Fatty acid derivatives isolated from *Petrosia* sponges.

A cyclitol derivative (**271**) was reported as a metabolite of Korean *Petrosia* sp. (Figure 20) [114]. This compound exhibited minimal cytotoxicity against a panel of human solid tumor cells with IC_50_ values around 10.0 μg/mL; however, HeLa extract-promoted in vitro DNA replication was inhibited by the treatment of **271** in a dose-dependent manner. Sphingolipid **272** was isolated from Red Sea *Petrosia* sp. and exhibited minimal cytotoxicity against MCF-7 and HepG2 cell lines with IC_50_ values around 20.0 μg/mL [69]. Brominated fatty acids (**273**–**277**) were isolated from the Caribbean *Petrosia* sp., and its structure was elucidated by mass spectrometry and chemical transformations, including deuteration with Wilkinson’s catalyst [115].

## 3. Discussion

**Figure 21:**
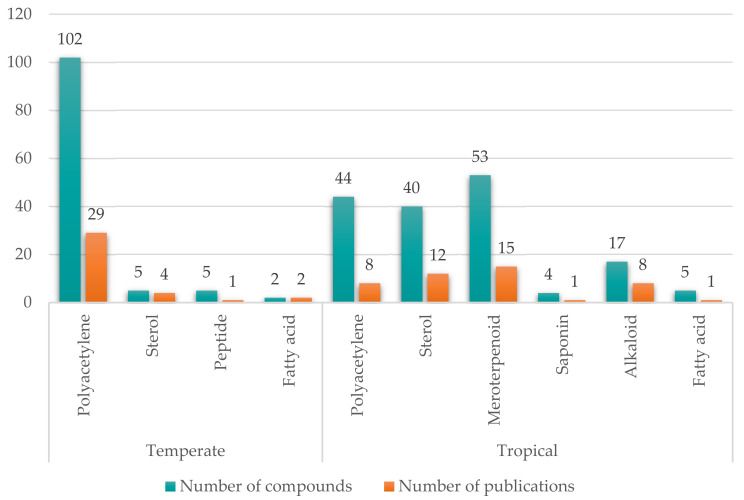
Number of compounds and publications for each type of *Petrosia* metabolites.

This review addressed 277 compounds isolated from *Petrosia* sponges from 87 peer-reviewed articles (Figure 21). The type of predominant metabolite depended on the geographical location of the source sponge. Polyacetylenes were the most frequently found in *Petrosia* sponges inhabiting temperate regions. Of the 36 research articles regarding the metabolites isolated from sponges collected in the Mediterranean Sea, Korean waters, or the temperate regions of Japan, 29 reported the isolation of polyacetylenes. More than 100 polyacetylenes have been isolated from temperate *Petrosia* sponges, and 44 polyacetylenes were reported as the metabolites of tropical *Petrosia* sponges.

Meroterpenoids and sterols were the most frequently found metabolites in tropical *Petrosia* sponges; 15 and 12 articles of the 50 papers on the metabolites of tropical *Petrosia* sponges report a total of 53 meroditerpenoids and 40 steroids, respectively. There are only five reported sterols isolated from *Petrosia* collected in Italy and Japan, and no reported meroterpenoids have been isolated from temperate *Petrosia* sponges. Alkaloids have also only been isolated from tropical *Petrosia* sponges. Seventeen alkaloids have been reported in eight publications regarding the *Petrosia* sponges collected in the Philippines, Indonesia, Papua-New Guinea, India, Belize, and Saudi Arabia.

Saponins and peptides have also been isolated from *Petrosia* sponges, but they are not very common. Four noriterpene glycosides of the sarasinoside class were isolated from the Indonesian *Petrosia* sp., and five depsipeptides were isolated from a Korean *Petrosia* sponge. Considering the scarcity of these compounds, their origins might be the sponge-associated microbes.

All metabolites isolated from *Petrosia* sponges from temperate and tropical regions are summarized in Table 1 and Table 2, respectively.

Considering that the actual producers of natural products isolated from sponges are the microbes associated therein in many cases [8,10], the latitudinal variation in metabolite composition of *Petrosia* sponges might be correlated with their associated microbial community. Genes involved in the biosynthesis of *Petrosia* metabolites have never been deduced until now; however, the biosynthetic gene clusters for analogous polyacetylenes, meroterpenoids, and terpenoids have been found from fungi, cyanobacteria, and actinomycetes. In addition, it has been reported for *P. ficiformis* that microbial symbiotic populations were more similar in genetically distinct individuals from the same location, than in genetically similar individuals from distant regions [116]. The factors which affects the structure of associated microbial community might be diverse and complex, as tropical and temperate seas have completely different ecosystems, not to mention physical factors, such as temperature and salinity. To better understand the factors that govern the composition of sponge metabolites, research to analyze the structure of associated microbial community of the sponges using various metagenomics tools should be conducted.

The biological activity most frequently investigated for *Petrosia* metabolites was cytotoxicity; most of the petrosiacetylenes showed growth inhibitory activity against human cancer cell lines. Research to better understand the ecological and pharmacological role of these compounds should be conducted. Biogenesis of *Petrosia* metabolites would be another significant topic in this field. This research would reveal the symbiotic relationships between marine sponges and microorganisms, and the strategies in biosynthesis that can be applied to the production of biologically-relevant molecules. In addition, the discovery of novel genes involved in the biosynthesis would provide potential bioengineering applications.

## Figures and Tables

**Table 1:** Metabolites isolated from *Petrosia* sponges in temperate region.

Compound	Examined Biological Activity	Species	Reference
* Polyacetylenes*			
* petroformynes*			
**1**–**4**	toxicity (fertilized sea urchin eggs, *A. salina*)	*P. ficiformis*	[28,29]
**1**–**15**, **18**–**20**, **22**–**24**	brine shrimp lethality	*P. ficiformis*	[30,31]
**16**, **17**, **21**, **25**	not reported	*P. ficiformis*	[32]
* petrosynol and petrosynone*			
**26**, **27**	toxicity (fertilized sea urchin eggs)	*Petrosia* sp.	[33,34]
**28**–**31**	toxicity (fertilized ascidan eggs), brine shrimp lethality	*Petrosia* sp.	[35]
* petroacetylene*			
**32**	inhibition of the blastulation of starfish embryo	*Petrosia* sp.	[36]
* petroformynes and neopetroformynes*			
**1**, **4**, **33**–**36**	cytotoxicity (P388)	*Petrosia* sp.	[38]
* durynes and miyakosynes*			
**37**–**42**	cytotoxicity (HeLa)	*Petrosia* sp.	[39]
**43**–**48**	cytotoxicity (HeLa)	*Petrosia* sp.	[42]
* petrosynes*			
**49**–**50**	not reported	*Petrosia* sp.	[43]
* corticatic acids*			
**51**–**53**	antifungal activity (*M. ramanniana*)	*P. corticata*	[44]
**54**–**55**	inhibition of GGTase I from *C. albicans*	*P. corticata*	[45]
* petroformynes and petroformynic acids*			
**1**, **4**, **56**, **57**	cytotoxicity (P388)	*P. corticata*	[37]
* petrocortynes and petrosiacetylenes*			
**58**–**60**, **84**–**87**	brine shrimp lethality, RNA-cleaving activity	*Petrosia* sp.	[46]
**61**–**65**	cytotoxicity (K562)	*Petrosia* sp.	[47]
**66**–**83**, **89**, **90**	cytotoxicity (A549, SK-OV-3, SK-MEL-2, XF498, HCT-15)	*Petrosia* sp.	[48,49,50,51]
**84**–**86**, **88**	cytotoxicity (A549, SK-OV-3, SK-MEL-2, XF498, HCT-15)	*Petrosia* sp.	[52]
**84**–**86**, **91**	cytotoxicity (NCI-H23, PC-3, ACHN, NUGC-3, HCT-15)	*Petrosia* sp.	[53]
* petroraspailynes, raspailynes and petrosynol*			
**92**–**102**	cytotoxicity (K562)	*Petrosia* sp.	[54]
* Sterols*			
**187**–**188**	not reported	*P. ficiformis*	[77,78,79]
**187**–**191**	not reported	*P. corticata*	[80]
* Peptides*			
* halicylindramides*			
**266**–**270**	antagonistic activity against human farnesoid X receptor	*Petrosia* sp.	[113]
* Fatty acid derivatives*			
**271**	cytotoxicity (A549, SK-OC-3, SK-MEL-2, XF498, HCT-15)	*Petrosia* sp.	[114]
**272**	cytotoxicity (MCF7, HepG2)	*Petrosia* sp.	[69]

**Table 2:** Metabolites isolated from *Petrosia* sponges in tropical region.

Compound	Examined Biological Activity	Species	Reference
* Polyacetylenes*			
* strongylodiols*			
**103**–**105**	cytotoxicity (MOLT-4, IMR-90, DLD-1)	*Petrosia* sp.	[56]
**106**–**112**	not reported	*Petrosia* sp.	[57]
* strongylotriols and pellynols*			
**113**–**117**	cytotoxicity (HeLa, K-562)	*Petrosia* sp.	[58]
* strongylodiols and petrosiols*			
**105**, **106**, **118**–**122**	induction of neuronal differentiation (PC-12)	*P. strongylata*	[59]
**123**–**131**	not reported	*Petrosia* sp.	[61]
**132**–**136**	cytotoxicity (NUGC)	*P. durissima*	[62]
* pellynols and petrosynoic acids*			
**115**, **137**–**144**	cytotoxicity (A2058, H522-T1, H460, IMR-90)	*Petrosia* sp.	[63]
* aztèquynols*			
**145**–**146**	cytotoxicity (KB)	*Petrosia* sp.	[64]
* Sterols*			
* strongylosterol*			
**147**	not reported	*P. durissima*	[65,66]
**148**–**151**	not reported	*P. durissima*	[67,68]
**152**–**158**	cytotoxicity (HepG2, MCF7)	*Petrosia* sp.	[69]
**159**–**174**	cytotoxicity (MOLT-3, HepG2, A549, HuCCA-1, HeLa, MDA-MB-231, MRC-5)	*Petrosia* sp.	[70]
* xestobergsterol A*			
**175**	not reported	*P.* cf. *cotignata*	[71]
* cotignasterol*			
**176**	not reported	*P. cotignata*	[72]
**177**–**179**	cytotoxicity (KB, LoVo)	*Petrosia* sp.	[73]
* orthoesterols*			
**180**–**182**	antiviral activity (FELV, PR8, A59)	*P. weinbergi*	[74]
* weinbersterols*			
**183**, **184**	antiviral activity (FELV, HIV)	*P. weinbergi*	[75]
* lembehsterols*			
**185**, **186**	inhibition of thymidine phosphorylase	*P. strongylata*	[76]
* Meroterpenoids*			
* strongylophorines*			
**192**–**194**	not reported	*P. durissima*	[83]
**195**-**199**	antimicrobial activity (*B. subtilis*, *S. aureus*)	*P. durissima*	[84]
**192**–**194**, **196**, **200**–**202**	cytotoxicity (NUGC)	*P. durissima*	[62,85]
**192**–**195**, **199**, **203**–**205**, **209**, **210**	cytotoxicity (HeLa)	*P. corticata*	[86]
**193**–**195**, **199**, **207**–**213**	inhibition of the maturation of starfish oocytes	*P. strongylata*	[87]
**193**, **194**, **199**, **209**, **211**, **214**, **215**	inhibition of protein tyrosine phosphatase 1B	*P. strongylata*	[88]
**193**-**195**, **199**, **203**, **207**, **208**, **214**, **215**	chymotrypsin-like activity of proteasome	*P. corticata*	[89]
**199**, **216**	cytotoxicity (MDA-231)	*P. corticata*	[90]
**193**–**195**, **217**	antimicrobial activity (*M. lueus*, *S. typhi*, *C. cucumerinum*), brine shrimp lethality	*Petrosia* sp.	[91]
**218**, **222**–**236**	inhibition of ubiquitin-specific protease 7	*P. alfiani*	[95]
**219**–**221**, **237**–**240**	inhibition of iron chelator-induced HIF-1, cytotoxicity (T47D, MDA-MB-231)	*P. alfiani*	[96]
* dihalenaquinolides*			
**241**, **242**	cytotoxicity (Hep3B)	*P. elastica*	[97]
* puupehenone*			
**243**	cytotoxicity (P388, A549, MCF7)	*P. hartmani*	[98]
* strongylin A*			
**244**	cytotoxicity (P388), antiviral activity (PR8)	*P. hartmani*	[100]
* Saponins*			
* sarasinosides*			
**245**–**248**	Cytotoxicity (Huh-7, A549)	*Petrosia* sp.	[101]
* Alkaloids*			
* petrosin*			
**249**	not reported	*P. seriata*	[105]
**250**–**253**	not reported	*Petrosia* sp.	[106]
**254**	not reported	*P. similis*	[107]
**255**–**257**	cytotoxicity (HCT116)	*Petrosia* sp.	[108]
* petrosamine*			
**258**	not reported	*Petrosia* sp.	[109]
**258**, **259**	inhibition of acetylcholinesterase	*Petrosia* sp.	[110]
* Nigricines*			
**260**–**263**	cytotoxicity (L5178Y)	*P. nigricans*	[111]
**264**, **265**	not reported	*P.* cf. *contignata*	[71]
* Fatty acid derivatives*			
**273**–**277**	not reported	*Petrosia* sp.	[115]

## References

[1] Lindequist U. (2016). Marine-derived pharmaceuticals—Challenges and opportunities. Biomol. Ther..

[2] Carroll A.R., Copp B.R., Davis R.A., Keyzers R.A., Prinsep M.R. (2019). Marine natural products. Nat. Prod. Rep..

[3] Carroll A.R., Copp B.R., Davis R.A., Keyzers R.A., Prinsep M.R. (2020). Marine natural products. Nat. Prod. Rep..

[4] Grosberg R.K., Vermeij G.J., Wainwright P.C. (2012). Biodiversity in water and on land. Curr. Biol..

[5] Proksch P. (2018). Chemical defence in marine ecosystems (From APR Volume 3). Annu. Plant Rev. Online.

[6] Hay M.E. (1996). Marine chemical ecology: What’s known and what’s next?. J. Exp. Mar. Biol. Ecol..

[7] Hentschel U., Hopke J., Horn M., Friedrich A.B., Wagner M., Hacker J., Moore B.S. (2002). Molecular evidence for a uniform microbial community in sponges from different cceans. Appl. Environ. Microbiol..

[8] Wang G. (2006). Diversity and biotechnological potential of the sponge-associated microbial consortia. J. Ind. Microbiol. Biotechnol..

[9] Hardoim C.C.P., Costa R., Araújo F.V., Hajdu E., Peixoto R., Lins U., Rosado A.S., Van Elsas J.D. (2009). Diversity of bacteria in the marine sponge *Aplysina fulva* in Brazilian coastal waters. Appl. Environ. Microbiol..

[10] Proksch P., Edrada R., Ebel R. (2002). Drugs from the seas—current status and microbiological implications. Appl. Microbiol. Biotechnol..

[11] Hu Y., Chen J., Hu G., Yu J., Zhu X., Lin Y., Chen S., Yuan J. (2015). Statistical research on the bioactivity of new marine natural products discovered during the 28 years from 1985 to 2012. Mar. Drugs.

[12] Leal M.C., Puga J., Serôdio J., Gomes N.C.M., Calado R. (2012). Trends in the discovery of new marine natural products from invertebrates over the last two decades—where and what are we bioprospecting?. PLoS ONE.

[13] Proksch P. (1994). Defensive roles for secondary metabolites from marine sponges and sponge-feeding nudibranchs. Toxicon.

[14] Ruzicka R., Gleason D.F. (2008). Latitudinal variation in spongivorous fishes and the effectiveness of sponge chemical defenses. Oecologia.

[15] Pawlik J.R., Loh T.-L., McMurray S.E., Finelli C.M. (2013). Sponge communities on Caribbean coral reefs are structured by factors that are top-down, not bottom-up. PLoS ONE.

[16] Poore A.G.B., Campbell A.H., Coleman R.A., Edgar G.J., Jormalainen V., Reynolds P.L., Sotka E.E., Stachowicz J.J., Taylor R.B., Vanderklift M.A. (2012). Global patterns in the impact of marine herbivores on benthic primary producers. Ecol. Lett..

[17] Floeter S.R., Behrens M.D., Ferreira C.E.L., Paddack M.J., Horn M.H. (2005). Geographical gradients of marine herbivorous fishes: Patterns and processes. Mar. Biol..

[18] Bakus G.J., Green G. (1974). Toxicity in sponges and holothurians: A geographic pattern. Science.

[19] Pawlik J., Chanas B., Toonen R., Fenical W. (1995). Defenses of Caribbean sponges against predatory reef fish. I. Chemical deterrency. Mar. Ecol. Prog. Ser..

[20] Burns E., Ifrach I., Carmeli S., Pawlik J., Ilan M. (2003). Comparison of anti-predatory defenses of Red Sea and Caribbean sponges. I. Chemical defense. Mar. Ecol. Prog. Ser..

[21] Becerro M.A., Thacker R.W., Turon X., Uriz M.J., Paul V.J. (2003). Biogeography of sponge chemical ecology: Comparisons of tropical and temperate defenses. Oecologia.

[22] Sacristan-Soriano O., Banaigs B., Becerro M.A. (2011). Relevant spatial scales of chemical variation in *Aplysina aerophoba*. Mar. Drugs.

[23] Rohde S., Gochfeld D.J., Ankisetty S., Avula B., Schupp P.J., Slattery M. (2012). Spatial variability in secondary metabolites of the Indo-Pacific sponge *Stylissa massa*. J. Chem. Ecol..

[24] Bayona L.M., Van Leeuwen G., Erol Ö., Swierts T., Van Der Ent E., De Voogd N.J., Choi Y.H. (2020). Influence of geographical location on the metabolic production of giant barrel sponges (*Xestospongia* spp.) revealed by metabolomics tools. ACS Omega.

[25] Castiello D., Cimino G., De Rosa S., De Stefano S., Sodano G. (1980). High molecular weight polyacetylenes from the nudibranch *Peltodoris actromaculata* and the sponge *Petrosia ficiformis*. Tetrahedron Lett..

[26] Cimino G., De Giulio A., De Rosa S., De Stefano S., Sodano G. (1985). Further high molecular weight polyacetylenes from the sponge *Petrosia ficiformis*. J. Nat. Prod..

[27] Cimino G., Crispino A., De Rosa S., De Stefano S., Sodano G. (1981). Polyacetylenes from the sponge *Petrosia ficiformis* found in dark caves. Cell. Mol. Life Sci..

[28] Cimino G., De Giulio A., De Rosa S., Di Marzo V. (1989). High molecular weight polyacetylenes from *Petrosia ficiformis*: Further structural analysis and biological activity. Tetrahedron Lett..

[29] Guo Y., Gavagnin M., Trivellone E., Cimino G. (1994). Absolute stereochemistry of petroformynes, high molecular polyacetylenes from the marine sponge *Petrosia ficiformis*. Tetrahedron.

[30] Cimino G., De Giulio A., De Rosa S., Di Marzo V. (1990). Minor bioactive polyacetylenes from *Petrosia ficiformis*. J. Nat. Prod..

[31] Guo Y., Gavagnin M., Trivellone E., Cimino G. (1995). Further structural studies on the petroformynes. J. Nat. Prod..

[32] Guo Y., Gavagnin M., Salierno C., Cimino G. (1998). Further petroformynes from both Atlantic and Mediterranean populations of the sponge *Petrosia ficiformis*. J. Nat. Prod..

[33] Fusetani N., Kato Y., Matsunaga S., Hashimoto K. (1983). Bioactive marine metabolites III. A novel polyacetylene alcohol, inhibitor of cell division in fertilized sea urchin eggs, from the marine sponge *Petrosia* sp.. Tetrahedron Lett..

[34] Fusetani N., Shiragaki T., Matsunaga S., Hashimoto K. (1987). Bioactive marine metabolites. 20. Petrosynol and petrosynone, antimicrobial C30 polyacetylenes from the marine sponge *Petrosia* sp.: Determination of the absolute configuration. Tetrahedron Lett..

[35] Ochi M., Ariki S., Tatsukawa A., Kotsuki H., Fukuyama Y., Shibata K. (1994). Bioactive polyacetylenes from the marine sponge *Petrosia* sp.. Chem. Lett..

[36] Ohta S., Ogawa T., Ohta E., Ikeuchi T., Kamemura K., Ikegami S. (2013). Petroacetylene, a new polyacetylene from the marine sponge *Petrosia solida* that inhibits blastulation of starfish embryos. Nat. Prod. Res..

[37] Okamoto C., Nakao Y., Fujita T., Iwashita T., Van Soest R.W.M., Fusetani N., Matsunaga S. (2007). Cytotoxic C47-polyacetylene carboxylic acids from a marine sponge *Pertrosia* sp.. J. Nat. Prod..

[38] Ueoka R., Ise Y., Matsunaga S. (2009). Cytotoxic polyacetylenes related to petroformyne-1 from the marine sponge *Petrosia* sp.. Tetrahedron.

[39] Hitora Y., Takada K., Okada S., Ise Y., Matsunaga S. (2011). (−)-Duryne and its homologues, cytotoxic acetylenes from a marine sponge *Petrosia* sp.. J. Nat. Prod..

[40] Wright A.E., McConnell O.J., Kohmoto S., Lui M.S., Thompson W., Snader K.M. (1987). Duryne, a new cytotoxic agent from the marine sponge Cribrochalinadura. Tetrahedron Lett..

[41] Gung B.W., Omollo A.O. (2008). Total synthesis of (+)- and (−)-duryne: A potent anticancer agent from the marine sponge cribrochalina Dura. Establishment of the central double bond geometry and the absolute configuration of the chiral centers. J. Org. Chem..

[42] Hitora Y., Takada K., Okada S., Matsunaga S. (2011). Miyakosynes A–F, cytotoxic methyl branched acetylenes from a marine sponge *Petrosia* sp.. Tetrahedron.

[43] Iguchi K., Kitade M., Kashiwagi T., Yamada Y. (1993). Structure and synthesis of petrosynes, new acetylenic enol ether glycerides from the Okinawan marine sponge of the genus *Petrosia*. J. Org. Chem..

[44] Li H.-Y., Matsunaga S., Fusteani N. (1994). Bioactive marine metabolites. corticatic acids A-C, antifungal acetylenic acids from the marine sponge, *Petrosia corticata*. J. Nat. Prod..

[45] Nishimura S., Matsunaga S., Shibazaki M., Suzuki K., Harada N., Naoki H., Fusetani N. (2002). Corticatic Acids D and E, polyacetylenic geranylgeranyltransferase type I inhibitors, from the marine sponge *Petrosia corticata*. J. Nat. Prod..

[46] Seo Y., Cho K.W., Rho J.-R., Shin J., Sim C.J. (1998). Petrocortynes and petrosiacetylenes, novel polyacetylenes from a sponge of the genus *Petrosia*. Tetrahedron.

[47] Shin J., Seo Y., Cho K.W. (1998). Five new polyacetylenes from a sponge of the genus *Petrosia*. J. Nat. Prod..

[48] Kim J.S., Lim Y.J., Im K.S., Jung J.H., Shim C.J., Lee C.O., Hong J., Lee H. (1999). Cytotoxic polyacetylenes from the marine sponge *Petrosia* sp.. J. Nat. Prod..

[49] Lim Y.J., Kim J.S., Im K.S., Jung J.H., Lee C.-O., Hong J., Kim D.-K. (1999). New Cytotoxic polyacetylenes from the marine sponge *Petrosia*. J. Nat. Prod..

[50] Lim Y.J., Park H.S., Im K.S., Lee C.-O., Hong J., Lee M.-Y., Kim D.-K., Jung J.H. (2001). Additional cytotoxic polyacetylenes from the marine sponge *Petrosia* species. J. Nat. Prod..

[51] Lim Y.J., Lee C.-O., Hong J., Kim D.-K., Im K.S., Jung J.H. (2001). Cytotoxic polyacetylenic alcohols from the marine sponge *Petrosia* species. J. Nat. Prod..

[52] Kim J.S., Im K.S., Jung J.H., Kim Y.-L., Kim J., Shim C.J., Lee C.-O. (1998). New bioactive polyacetylenes from the marine sponge *Petrosia* sp.. Tetrahedron.

[53] Lee Y.-J., Yoo S.-J., Kang J.S., Yun J., Shin H.J., Lee J.S., Lee H.-S. (2013). Cytotoxic petrosiacetylenes from the marine sponge *Petrosia* sp.. Lipids.

[54] Seo Y., Cho K.W., Lee H.-S., Rho J.-R., Shin J. (1999). New acetylenic enol ethers of glycerol from the sponge *Petrosia* sp.. J. Nat. Prod..

[55] Guella G., Mancini I., Pietra F. (1987). Raspailynes, novel long-chain acetylenic enol ethers of glycerol from the marine sponges *Raspailia pumila* and *Raspailia ramosa*. Helv. Chim. Acta.

[56] Watanabe K., Tsuda Y., Yamane Y., Takahashi H., Iguchi K., Naoki H., Fujita T., Van Soest R.W. (2000). Strongylodiols A, B and C, new cytotoxic acetylenic alcohols isolated from the Okinawan marine sponge of the genus *Strongylophora* as each enantiomeric mixture with a different ratio. Tetrahedron Lett..

[57] Watanabe K., Tsuda Y., Hamada M., Omori M., Mori G., Iguchi K., Naoki H., Fujita T., Van Soest R.W.M. (2005). Acetylenic strongylodiols from a *Petrosia* (*Strongylophora*) Okinawan marine sponge. J. Nat. Prod..

[58] Gabriel A.F., Li Z., Kusuda R., Tanaka C., Miyamoto T. (2015). Six new polyacetylenic alcohols from the marine sponges *Petrosia* sp. and *Halichondria* sp.. Chem. Pharm. Bull..

[59] Horikawa K., Yagyu T., Yoshioka Y., Fujiwara T., Kanamoto A., Okamoto T., Ojika M. (2013). Petrosiols A–E, neurotrophic diyne tetraols isolated from the Okinawan sponge *Petrosia strongylata*. Tetrahedron.

[60] Higashibayashi S., Czechtizky W., Kobayashi Y., Kishi Y. (2003). Universal NMR databases for contiguous polyols. J. Am. Chem. Soc..

[61] Watanabe K., Mori G., Iguchi K., Suzuki M., Van Soest R.W.M. (2007). Nine acetylenic alcohols isolated from the Okinawan marine sponge of the genus *Petrosia* (*Strongylophora*). Nat. Prod. Res..

[62] Shen Y.-C., Prakash C.V.S. (2000). Two new acetylenic derivatives and a new meroditerpenoid from a Taiwanese marine sponge *Strongylophora durissima*. J. Nat. Prod..

[63] Mejia E.J., Magranet L.B., De Voogd N.J., TenDyke K., Qiu D., Shen Y.Y., Zhou Z., Crews P. (2012). Structures and cytotoxic evaluation of new and known acyclic ene-ynes from an American Samoa *Petrosia* sp. sponge. J. Nat. Prod..

[64] Guerriero A., Debitus C., Laurent D., D’Ambrosio M., Pietra F. (1998). Aztequynol A, the first clearly defined, C-branched polyacetylene and the analog aztequynol B. Isolation from the tropical marine sponge *Petrosia* sp.. Tetrahedron Lett..

[65] Bortolotto M., Braekman J.C., Daloze D., Tursch B. (1978). Chemical studies of marine invertebrates. XXXVI. Strongylosterol, a novel C-30 sterol from the sponge *Strongylophora durissima* Dendy. Bull. Soc. Chim. Belg..

[66] Theobald N., Djerassi C. (1978). Determination of the absolute configuration of stelliferasterol and strongylosterol—Two marine sterols with “extended” side chains. Tetrahedron Lett..

[67] Li L.N., Djerassi C. (1981). Minor and trace sterols in marine invertebrates. 23. Xestospongesterol and isoxestospongesterol—First examples of quadruple biomethylation of the sterol side chain. J. Am. Chem. Soc..

[68] Li L.N., Djerassi C. (1981). Minor and trace sterols in marine invertebrates. 30. Isolation, structure elucidation, and partial synthesis of 26-methylstrongylosterol and 28-methylxestosterol, two marine sterols arising by a novel quadrupole biomethylation sequence. Tetrahedron Lett..

[69] Abdel-Lateff A., Alarif W.M., Asfour H.Z., Ayyad S.-E.N., Khedr A., Badria F.A., Al-Lihaibi S.S. (2014). Cytotoxic effects of three new metabolites from Red Sea marine sponge, *Petrosia* sp.. Environ. Toxicol. Pharmacol..

[70] Pailee P., Mahidol C., Ruchirawat S., Prachyawarakorn V. (2017). Sterols from Thai marine sponge *Petrosia* (*Strongylophora*) sp. and their cytotoxicity. Mar. Drugs.

[71] Sperry S., Crews P. (1998). Dihydrotubastrines: Phenethylguanidine analogs from the Indo-Pacific marine sponge *Petrosia* cf. contignata. J. Nat. Prod..

[72] Burgoyne D.L., Andersen R.J., Allen T.M. (1992). Contignasterol, a highly oxygenated steroid with the unnatural 14β configuration from the marine sponge *Petrosia contignata* Thiele, 1899. J. Org. Chem..

[73] Corgiat J.M., Scheuer P.J., Steiner J.L.R., Clardy J. (1993). Three pregnane-10,2-carbolactones from a sponge, *Strongylophora* sp.. Tetrahedron.

[74] Koehn F.E., Gunasekera M., Cross S.S. (1991). New antiviral sterol disulfate ortho esters from the marine sponge *Petrosia weinbergi*. J. Org. Chem..

[75] Sun H.H., Gross S.S., Gunasekera M., Koehn F.E. (1991). Weinbersterol disulfates A and B, antiviral steroid sulfates from the sponge petrosia weinbergi. Tetrahedron.

[76] Aoki S., Naka Y., Itoh T., Furukawa T., Rachmat R., Akiyama S.-I., Kobayashi M. (2002). Lembehsterols A and B, novel sulfated sterols inhibiting thymidine phosphorylase, from the marine sponge *Petrosia strongylata*. Chem. Pharm. Bull..

[77] Sica D., Zollo F. (1978). Petrosterol, the major sterol with a cyclopropane side chain in the sponge *Petrosia ficiformis*. Tetrahedron Lett..

[78] Mattia C., Mazzarella L., Puliti R., Sica D., Zollo F. (1978). X-ray crystal structure determination of petrosterol p-bromobenzoate. A revision. Tetrahedron Lett..

[79] Khalil M.W., Durham L.J., Djerassi C., Sica D. (1980). Minor and trace sterols in marine invertebrates. 15. Ficisterol (23-ethyl-24-methyl-27-norcholesta-5,25-dien-3.beta.-ol). A biosynthetically unprecedented sterol from the marine sponge *Petrosia ficiformis*. J. Am. Chem. Soc..

[80] Umeyama A., Ito S., Yoshigaki A., Arihara S. (2000). Two new 26,27-cyclosterols from the marine sponge *Strongylophora corticata*. J. Nat. Prod..

[81] Li L.N., Li H.T., Lang R.W., Itoh T., Sica D., Djerassi C. (1982). Minor and trace sterols in marine invertebrates. 31. Isolation and structure elucidation of 23H-isocalysterol, a naturally occurring cyclopropene. Some comparative observations on the course of hydrogenolytic ring opening of steroidal cyclopropenes and cyclopropanes. J. Am. Chem. Soc..

[82] Menna M., Imperatore C., D’Aniello F., Aiello A. (2013). Meroterpenes from marine invertebrates: Structures, occurrence, and ecological implications. Mar. Drugs.

[83] Braekman J.C., Daloze D., Hulot G., Tursch B., Declercq J.P., Germain G., Van Meerssche M. (1978). Chemical studies of marine invertebrates. XXXVII. Three novel meroditerpenoids from the sponge *Strongylophora durissima*. Bull. Soc. Chim. Belg..

[84] Salva J., Faulkner D.J. (1990). Metabolites of the sponge *Strongylophora durissima* from Maricaban Island, Philippines. J. Org. Chem..

[85] Shen Y.-C., Hung M.-C., Prakash C.V.S., Wang J.-J. (2000). New meroditerpenoids from a Taiwanese marine sponge *Strongylophora durissima*. J. Chin. Chem. Soc..

[86] Hoshino A., Mitome H., Miyaoka H., Shintani A., Yamada Y., Van Soest R.W.M. (2003). New strongylophorines from the Okinawan marine sponge *Petrosia* (*Strongylophora*) *corticata*. J. Nat. Prod..

[87] Liu H., Namikoshi M., Akano K., Kobayashi H., Nagai H., Yao X. (2005). Seven new meroditerpenoids, from the marine sponge *Strongylophora strongylata*, that inhibited the maturation of starfish oocytes. J. Asian Nat. Prod. Res..

[88] Lee J.-S., Abdjul D.B., Yamazaki H., Takahashi O., Kirikoshi R., Ukai K., Namikoshi M. (2015). Strongylophorines, new protein tyrosine phosphatase 1B inhibitors, from the marine sponge *Strongylophora strongilata* collected at Iriomote Island. Bioorganic Med. Chem. Lett..

[89] Noda A., Sakai E., Kato H., Losung F., Mangindaan R.E., De Voogd N.J., Yokosawa H., Tsukamoto S. (2015). Strongylophorines, meroditerpenoids from the marine sponge *Petrosia corticata*, function as proteasome inhibitors. Bioorg. Med. Chem. Lett..

[90] Warabi K., McHardy L.M., Matainaho L., Van S.R., Roskelley C.D., Roberge M., Andersen R.J. (2004). Strongylophorine-26, a new meroditerpenoid isolated from the marine sponge *Petrosia* (*Strongylophora*) *corticata* that exhibits anti-invasion activity. J. Nat. Prod..

[91] Balbin-Oliveros M., Edrada R.A., Proksch P., Wray V., Witte L., Van Soest R.W.M. (1998). A new meroditerpenoid dimer from an undescribed Philippine marine sponge of the genus *Strongylophora*. J. Nat. Prod..

[92] Roll D.M., Scheuer P.J., Matsumoto G.K., Clardy J. (1983). Halenaquinone, a pentacyclic polyketide from a marine sponge. J. Am. Chem. Soc..

[93] Nakamura H., Kobayashi J.i., Kobayashi M., Ohizumi Y., Hirata Y. (1985). Physiologically active marine natural products from *Porifera*. VII. Xestoquinone. A novel cardiotonic marine natural product isolated from the Okinawan sea sponge *Xestospongia sapra*. Chem. Lett..

[94] Schmitz F.J., Bloor S.J. (1988). Xesto- and halenaquinone derivatives from a sponge, *Adocia* sp., from Truk lagoon. J. Org. Chem..

[95] Tanokashira N., Kukita S., Kato H., Nehira T., Angkouw E.D., Mangindaan R.E., de Voogd N.J., Tsukamoto S. (2016). Petroquinones: Trimeric and dimeric xestoquinone derivatives isolated from the marine sponge *Petrosia alfiani*. Tetrahedron.

[96] Du L., Mahdi F., Datta S., Jekabsons M.B., Zhou Y.-D., Nagle D.G. (2012). Structures and mechanisms of antitumor agents: Xestoquinones uncouple cellular respiration and disrupt HIF signaling in human breast tumor cells. J. Nat. Prod..

[97] Shen Y.C., Prakash C.V.S., Guh J.-H. (2004). New pentacyclic polyketide dimeric peroxides from a Taiwanese marine sponge *Petrosia elastica*. Tetrahedron Lett..

[98] Kohmoto S., McConnell O.J., Wright A., Koehn F., Thompson W., Lui M., Snader K.M. (1987). Puupehenone, a cytotoxic metabolite from a deep water marine sponge, *Stronglyophora hartmani*. J. Nat. Prod..

[99] Ravi B.N., Perzanowski H.P., Ross R.A., Erdman T.R., Scheuer P.J., Finer J., Clardy J. (1979). Recent research in marine natural products: The puupehenones. Pure Appl. Chem..

[100] Wright A.E., Rueth S.A., Cross S.S. (1991). An antiviral sesquiterpene hydroquinone from the marine sponge *Strongylophora hartmani*. J. Nat. Prod..

[101] Maarisit W., Yamazaki H., Kanno S.-I., Tomizawa A., Rotinsulu H., Wewengkang D.S., Sumilat D.A., Ukai K., Kapojos M.M., Namikoshi M. (2017). A tetramic acid derivative with protein tyrosine phosphatase 1B inhibitory activity and a new nortriterpene glycoside from the Indonesian marine sponge *Petrosia* sp.. Bioorganic Med. Chem. Lett..

[102] Espada A., Jiménez C., Rodriguez J., Crews P., Riguera R. (1992). Sarasinosides D-G: Four new triterpenoid saponins from the sponge asteropus sarasinosum. Tetrahedron.

[103] Santalova E.A., Denisenko V.A., Dmitrenok P.S., Berdyshev D.V., Stonik V.A. (2006). Two new sarasinosides from the sponge *Melophlus Sarasinorum*. Nat. Prod. Commun..

[104] Lee J.-H., Jeon J.-E., Lee Y.-J., Lee H.-S., Sim C.J., Oh K.-B., Shin J. (2012). Nortriterpene glycosides of the sarasinoside class from the sponge *Lipastrotethya* sp.. J. Nat. Prod..

[105] Braekman J.C., Daloze D., Macedo de Abreu P., Piccinni-Leopardi C., Germain G., Van Meerssche M. (1982). A novel type of bisquinolizidine alkaloid from the sponge *Petrosia seriata*. Tetrahedron Lett..

[106] Kobayashi M., Rao S.R., Chavakula R., Sarma N.S. (1994). Mimosamycin, 4-aminomimosamycin and 7-amino-7-demethoxymimosamycin from the *Petrosia* sp. of sponge. J. Chem. Res. Synop..

[107] Ramesh P., Reddy N.S., Venkateswarlu Y. (1999). A new 1,2-dihydroisoquinoline from the sponge *Petrosia similis*. J. Nat. Prod..

[108] Sandoval I.T., Davis R.A., Bugni T.S., Concepcion G.P., Harper M.K., Ireland C.M. (2004). Cytotoxic isoquinoline quinones from sponges of the genus *Petrosia*. Nat. Prod. Res..

[109] Molinski T.F., Fahy E., Faulkner D.J., Van Duyne G.D., Clardy J. (1988). Petrosamine, a novel pigment from the marine sponge *Petrosia* sp.. J. Org. Chem..

[110] Nukoolkarn V.S., Saen-Oon S., Rungrotmongkol T., Hannongbua S., Ingkaninan K., Suwanborirux K. (2008). Petrosamine, a potent anticholinesterase pyridoacridine alkaloid from a Thai marine sponge *Petrosia* sp.. Bioorg. Med. Chem..

[111] Ashour M., Edrada-Ebel R., Ebel R., Wray V., Van Soest R., Proksch P. (2008). New purine derivatives from the marine sponge *Petrosia nigricans*. Nat. Prod. Commun..

[112] Ryuichi S., Tatsuo H. (1987). Tubastrine, a new guanidinostyrene from the coral *Tubastrea aurea*. Chem. Lett..

[113] Hahn D., Kim H., Yang I., Chin J., Hwang H., Won D.H., Lee B., Nam S.-J., Ekins M., Choi H. (2015). The halicylindramides, farnesoid X receptor antagonizing depsipeptides from a *Petrosia* sp. marine sponge collected in Korea. J. Nat. Prod..

[114] Kim D.-K., Lim Y.J., Kim J.S., Park J.H., Kim N.D., Im K.S., Hong J., Jung J.H. (1999). A cyclitol derivative as a replication inhibitor from the marine sponge *Petrosia* sp.. J. Nat. Prod..

[115] Carballeira N.M., Shalabi F. (1993). Novel brominated phospholipid fatty acids from the Caribbean sponge *Petrosia* sp.. J. Nat. Prod..

[116] Burgsdorf I., Haber M., Steindler L., Erwin P.M., Lopez-Legentil S., Cerrano C., Frenk S. (2014). Biogeography rather than association with cyanobacteria structures symbiotic microbial communities in the marine sponge *Petrosia ficiformis*. Front. Microbiol..

